# Piscine Reovirus: Genomic and Molecular Phylogenetic Analysis from Farmed and Wild Salmonids Collected on the Canada/US Pacific Coast

**DOI:** 10.1371/journal.pone.0141475

**Published:** 2015-11-04

**Authors:** Ahmed Siah, Diane B. Morrison, Elena Fringuelli, Paul Savage, Zina Richmond, Robert Johns, Maureen K. Purcell, Stewart C. Johnson, Sonja M. Saksida

**Affiliations:** 1 British Columbia Centre for Aquatic Health Sciences, Campbell River, British Columbia, Canada; 2 Marine Harvest Canada, Campbell River, British Columbia, Canada; 3 Veterinary Sciences Division, AFBI Stormont, Stoney Road, Belfast, United Kingdom; 4 US Geological Survey, Western Fisheries Research Center, Seattle, WA, United States of America; 5 Pacific Biological Station, Nanaimo, British Columbia, Canada; Ecole normale superieure de Lyon, FRANCE

## Abstract

Piscine reovirus (PRV) is a double stranded non-enveloped RNA virus detected in farmed and wild salmonids. This study examined the phylogenetic relationships among different PRV sequence types present in samples from salmonids in Western Canada and the US, including Alaska (US), British Columbia (Canada) and Washington State (US). Tissues testing positive for PRV were partially sequenced for segment S1, producing 71 sequences that grouped into 10 unique sequence types. Sequence analysis revealed no identifiable geographical or temporal variation among the sequence types. Identical sequence types were found in fish sampled in 2001, 2005 and 2014. In addition, PRV positive samples from fish derived from Alaska, British Columbia and Washington State share identical sequence types. Comparative analysis of the phylogenetic tree indicated that Canada/US Pacific Northwest sequences formed a subgroup with some Norwegian sequence types (group II), distinct from other Norwegian and Chilean sequences (groups I, III and IV). Representative PRV positive samples from farmed and wild fish in British Columbia and Washington State were subjected to genome sequencing using next generation sequencing methods. Individual analysis of each of the 10 partial segments indicated that the Canadian and US PRV sequence types clustered separately from available whole genome sequences of some Norwegian and Chilean sequences for all segments except the segment S4. In summary, PRV was genetically homogenous over a large geographic distance (Alaska to Washington State), and the sequence types were relatively stable over a 13 year period.

## Introduction

Heart and skeletal muscle inflammation (HSMI) is an infectious disease which was first diagnosed by histology in 1999 in farmed Atlantic salmon *Salmo salar* from Norway [1, 2]. To date, this disease has only been reported in farmed Atlantic salmon in Norway, and from a single outbreak in farmed Atlantic salmon in Scotland [3]. A decade after HSMI was first detected, 454 pyrosequencing of heart tissue obtained from Atlantic Salmon showing signs of HSMI, as well as cardiomyopathy syndrome (CMS), resulted in the identification of Piscine reovirus (PRV) and the assembly of its genomic sequence [4]. Although PRV was proposed to be the causal agent of HSMI [4, 5], recent studies using molecular diagnostics have found PRV to be present in wild and farmed Atlantic salmon that are clinically healthy, as well as those suffering from HSMI [5, 6].

PRV belongs to the family of *Reoviridae*; it is a double stranded non-enveloped RNA virus with 10 nucleic acid segments L1, L2, L3, M1, M2, M3, S1, S2, S3, S4 encoding for λ3, λ2/p11, λ1, μ2, μ1, μNS, σ3/p13, σ 2/p8, σNS, σ1 proteins respectively [4, 7]. PRV differs from the other reoviruses by its 5’ terminal nucleotides and the proteins σ3/p13 and σ2/p8 encoded by the bicistronic genes S1 and S2 [4, 7, 8]. Transfection of fish cell lines with *E*. *coli* expressing p13 protein indicated that p13 is not a fusion-associated small transmembrane (FAST) protein present in some orthoreoviruses and aquareoviruses [8], as suggested previously by others [4], but an integral membrane protein localized in Golgi-like structure with cytotoxicity features [7, 9]. These features suggest that PRV could represent a new genus in the *Reoviridae* family that is more closely related to orthoreoviruses than aquareoviruses [8, 9]. However, the taxonomy of PRV is still under investigation and the virus remains unclassified.

PRV is known to occur in a wide variety of salmon species on the Pacific Coast of North America, a region where HSMI has never been reported [10, 11]. Kibenge et al (2013) [10] sequenced the whole genome of PRV positive samples (samples #358 and #371) obtained from samples of farmed Atlantic salmon purchased from a retailer in British Columbia (B.C.), as well as partial sequences of other genomic regions from wild Cutthroat trout (*Oncorhynchus clarkii*), farmed steelhead trout (*Oncorhynchus mykiss*), wild Chum salmon (*Oncorhynchus keta*) from British Columbia and farmed Atlantic salmon from Chile. Their phylogenetic analysis of segment S1 grouped Norwegian PRV strains into a single genotype of two sub-genotypes (la and l b) with B.C. strains grouping with sub-genotype la and Chilean strains with sub-genotype Ib. These authors suggested that the virus present in B.C. and Chile diverged from Norwegian viruses in 2007 and 2008 (±1 year), respectively. More recently, the screening of 363 archived and 916 fresh-frozen salmonids tissues collected in B.C. and Alaska between 1974 and 2013 identified the presence of PRV in salmon originating from both of these areas with the earliest detection being from a sample of wild Steelhead Trout collected in 1977 [11].

A recent study that used PRV as a model organism to investigate the viral transmission between wild and farmed salmonids showed a high level of exchange of the virus between the wild and farmed salmonid populations in Norway [12]. The phylogenetic analysis from this study identified five major groups and several clusters within Norway [12].

In Canada/US Pacific coast, genetic diversity of PRV RNA sequences is still unknown. Taking advantage from recent surveys in Washington State, British Columbian and Alaskan waters [11], this study aims at investigating both the occurrence and genetic diversity of PRV sequences isolated from wild and farmed fish collected in these regions. Particularly, it was of interest to focus on geographic divergence of partial segment S1 including ORF p13 protein within North West Coast of America and in comparison to European and South American sequences mainly from Norway and Chile available in GenBank. We initially targeted the PRV S1 segment; this segment has been phylogenetically informative in previous studies [10]. In this study, 71 sequences were isolated from wild and farmed fish collected from 21 different locations over a 13 year period. In addition, we analyzed the genome sequences on selected PRV positive samples based on their geographic localization in order to examine the genomic diversity within these waters in comparison to previously reported PRV genome sequences [4, 10].

## Materials and Methods

### Sample collection

**Fig 1:**
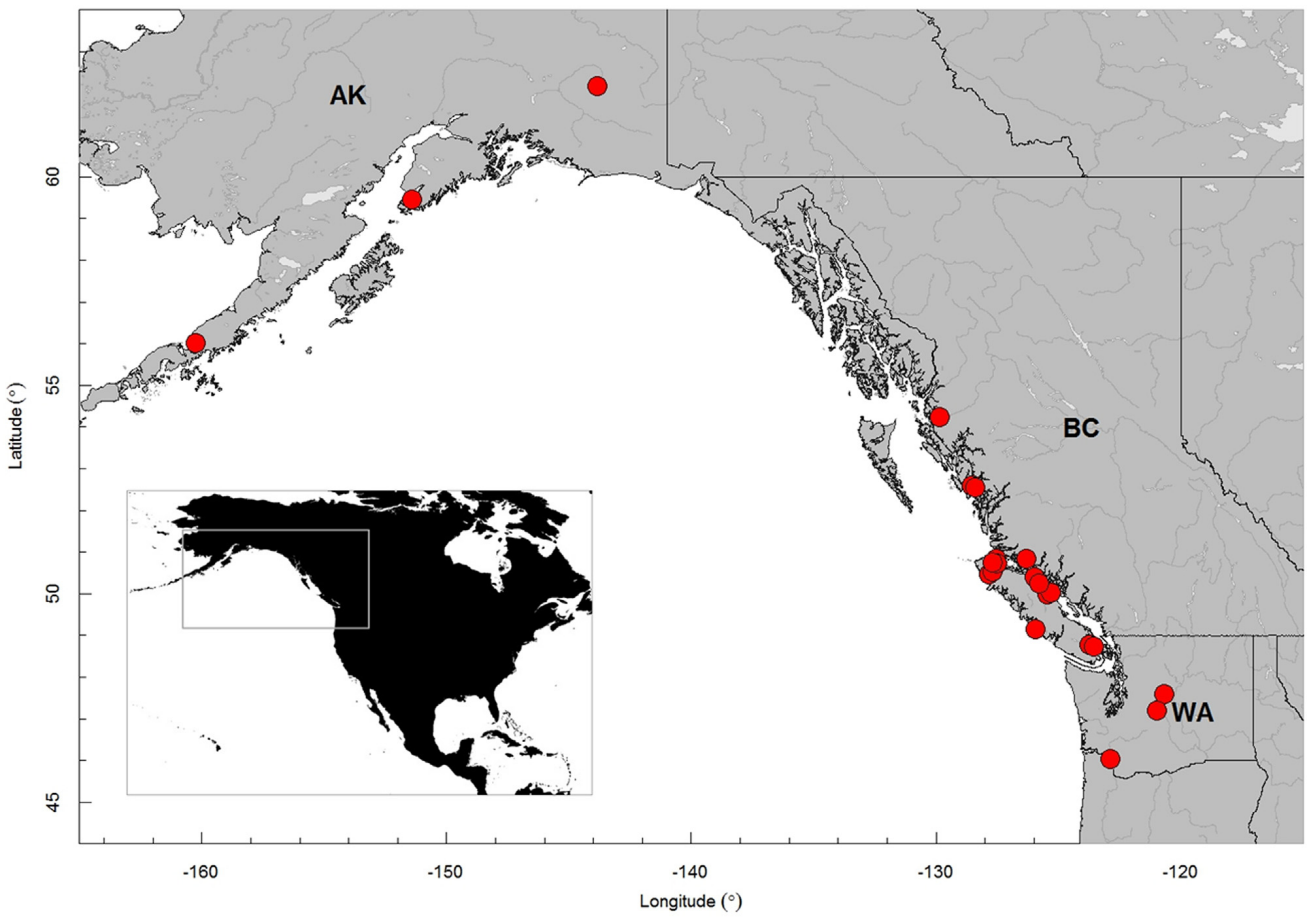
Map of Canada/US Pacific coast showing the geographic localisations where the salmonids were sampled. AK: Alaska (United States of America); BC: British Columbia (Canada); WA: Washington State (United States of America).

Tissue samples were collected from wild and farmed salmonids from different locations on the Canada/US Pacific coast. These samples were collected during recent surveys for PRV in B.C. and Alaska [11] and from fish health survey work in Washington State (Fig 1).

Washington samples were collected by fish health professionals as part of routine fish health screenings (e.g. as part of a veterinary record) and permits were obtained from the managing agencies (Washington Department of Fish and Wildlife Service or US Fish and Wildlife Service). Fish were euthanized in compliance with AVMA standards; specifically, juvenile fish were euthanized by an intentional overdose of tricaine methanesulfonate and adult fish were euthanized by blunt force trauma to the cranium followed by exsanguination or cervical transection. Canadian, Alaskan hatchery and seawater farmed fish were euthanized by a lethal dose of the anesthetic (tricaine methanesulfonate) following the methods set out in the Fisheries and Oceans Canada, Pacific Region Animal Care Committee Standard Operating Procedures as well as BCVMA/CVMA/AAVBC and RSPCA United Kingdom approved Atlantic salmon standard methodology. Wild fish from British Columbia were sampled by recreational sportfishers who killed the fish by percussion. Tissues had been removed by the sportfishers, placed in plastic bags for transfer to the onsite sampling fish health professional team.

Samples of wild and hatchery salmon collected in Canadian waters were collected under scientific licence pursuant to Section 52 of the Fisheries (General) Regulations issued by the Regional Director General, Pacific Region Department of Fisheries and Oceans (DFO), Canada. These licences allow capture of fish for scientific purposes in Canadian Fisheries waters.

According to Canadian Council on Animal Care work not requiring protocols or inclusion in animal use inventories includes: Fishes being lethally sampled under government or other regulatory mandate for established fish inspection procedures, abundance estimates, and other population parameters required for assessing stocks and for routine monitoring of contamination/toxin levels and disease. This work falls within this category.

From these collections, representative PRV positive samples were selected for sequencing based on their real-time reverse-transcriptase PCR Ct values (Ct<30). This Ct value cut-off point was chosen because samples with Ct values higher than 30 do not amplify well by conventional PCR and products typically yield sequences of poor quality.

Aseptic technique was used to obtain fresh fish tissues. Briefly, 1–3 grams of tissue were collected using dissection tools cleaned with 10% bleach, rinsed in 70% ethanol and flamed. A new set of dissection tools was used for each fish. Tissue samples were placed in sterile Whirl-Pak bags (25 ml), placed on ice immediately after dissection and frozen within an hour at -20°C before shipping. Samples were shipped frozen and upon arrival at the laboratory, they were transferred while frozen into RNase-free vials and stored at -80°C until analysis. Sections of paraffin embedded archived salmon tissues that had previously tested positive for PRV were provided by the Animal Health Centre, Ministry of Agriculture (Abbotsford, BC). These samples were prepared for RNA extraction as described in Marty et al (2014) [11].

### RNA extraction and reverse transcriptase polymerase chain reaction (RT-PCR)

RNA was extracted from approximately 30 mg of frozen tissues using Qiagen RNeasy Mini kit according to the manufacturer’s recommendations (Qiagen, ON, Canada). Total RNA was eluted in 50 μL of RNAse-free water. RNA was extracted from paraffin embedded tissues using RNeasy FFPE Kit (Qiagen, ON, Canada) according to the manufacturer’s recommendations. Total extracted RNA was also eluted in 50 μL of RNAse-free water (Qiagen, ON, Canada). Extracted RNA samples were stored in -80°C until used.

To obtain DNA for sequencing, PRV segment S1 was amplified from total RNA using the primer sets described in Kibenge et al (2013) [10] (Table 1). RT-PCR was performed using Qiagen One Step RT-PCR kit (Qiagen, ON, Canada). Each RT-PCR reaction (20 μL total volume) included 625 nM of each reverse and forward primer and 2 μL of total extracted RNA. Amplifications were performed using Mastercycler Eppendorf (Fisher Scientific, ON, Canada) under the following conditions: RT step—50°C for 40 min and PCR steps—denaturation at 95°C for 10 min; 40 cycles of 95°C for 30 sec, 54°C for 30 sec and 72°C for 70 sec followed by a final extension at 72°C for 10 min. In the case of paraffin embedded tissues, 14 different primer sets were used for segment S1 amplification (Table 1). RT-PCR was performed as described above. However, annealing temperatures were changed to match the primer set being used. Ethidium bromide stained PCR products were separated using a 1.8% agarose gel and visualized using Molecular Imager Chemidoc^™^ XRS^+^ with Image Lab software (Biorad, ON, Canada).

**Table 1:** Primer sequences used for Reverse Transcriptase—Polymerase Chain Reaction and sequencing of partial segment S1 of piscine reovirus. Ta is the annealing temperature used for each set of primers.

Name	Sequences	Positions	Direction	Ta (°C)	Source
PRV-S1-F1	GATAAAGACTTCTGTACGTGAAAC	1–24	Forward	54	Kibenge et al [10]
PRV-S1-R1	GATGAATAAGACCTCCTTCC	1,062–1,081	Reverse	54	Kibenge et al [10]
S1PRV14F	GTACGTGAAACCCAAATGGCG	14–34	Forward	62	This study
S1PRV295R	CCAATCTCCGGGTGCAGAAT	276–295	Reverse	62	This study
S1PRV800F	CGAAGGATGTGCTATTCAGGC	800–820	Forward	62	This study
S1PRV1070R	CCTCCTTCCCCCTCAGAC	1,053–1,070	Reverse	62	This study
S1PRV287F1	GGAGATTGGACATGGAGTG	287–305	Forward	54	This study
S1PRV542R1	GATTTCAACTCGTCAGGG	535–552	Reverse	54	This study
S1PRV273F2	ATCATTCTGCACCCGGAG	273–290	Forward	54	This study
S1PRV569R2	TTGAATAGTCAGGGAAAGA	551–569	Reverse	54	This study
S1PRV541F	CGAGTTGAAATCTTTCCCTG	541–560	Forward	54	This study
S1PRV838R	GATGATGTTGGCTGGCATGCC	818–838	Reverse	54	This study
S1PRV541F	CGAGTTGAAATCTTTCCCTG	541–560	Forward	62	This study
S1PRV840R	TTGATGATGTTGGCTGGC	823–840	Reverse	62	This study
S1PRV270F	GGGATCATTCTGCACCCGG	270–288	Forward	54	This study
S1PRV562R	GTCAGGGAAAGATTTCAACTCG	541–562	Reverse	54	This study
S1PRV287F1	GGAGATTGGACATGGAGTG	287–305	Forward	54	This study
S1PRV569R2	TTGAATAGTCAGGGAAAGA	551–569	Reverse	54	This study
S1PRV12F1	CCTCTGGAGACCTTGGCTTG	495–514	Forward	60	This study
S1PRV244R	CAGGTACATCGCGATGGGAA	708–727	Reverse	60	This study
S1PRV25F	CCGCAAATCAGTTCCCACAC	657–676	Forward	60	This study
S1PRV238R	ATTCCAGAGAGCGCCATTCC	818–838	Reverse	60	This study
S1PRV56F	CATTGAAGCTAAGCGACGCC	439–458	Forward	60	This study
S1PRV246R	CAGTCTTCTGCTCCACTGGG	610–629	Reverse	60	This study
S1PRV12F2	ATCAATGCGATGGCGAAACG	246–286	Forward	60	This study
S1PRV224R	GGCGTCGCTTAGCTTCAATG	708–727	Reverse	60	This study
S1PRV33F	TGTGGGATCATTCTGCACCC	267–286	Forward	60	This study
S1PRV6F	CATTGAAGCTAAGCGACGCC	439–458	Forward	60	This study
S1PRV197R	CCAGTCTTCTGCTCCACTGG	611–630	Reverse	60	This study

### Sanger Sequencing and analysis

PRV segment S1 RT-PCR products were sequenced in both directions by a commercial sequencing service (Macrogen, Rockville, MD, US). PCR products were purified using QIAquick PCR Purification Kit (Qiagen, ON, Canada) and sequencing was carried out using the BigDye Terminator version 3.1 (Life Technologies, NY, US) and ABI 3730 Genetic Analyzer (Life Technologies, NY, US). Sequencing was performed using primers described in Table 1. Sequences were trimmed, assembled and inspected visually using Geneious version 6.1. The appropriate IUPAC (International Union of Pure and Applied Chemistry) code was applied in the case of base call ambiguities.

### Next-generation sequencing

Total RNA extracted from samples BCJ31915_13, BCJ19943_13 and WSKFH12_14 (Table 2) were submitted to ACGT Inc. (IL, US) for next generation sequencing (NGS). RNA was evaluated for quantity and quality using an Agilent 2100 Bioanalyzer (Agilent, CA, US) RNA 6000 chip. Sequencing libraries were prepared using the TruSeq mRNA sample prep kit. Mitochondrial ribosomal RNA sequences were removed using RiboZero Gold Kit prior to cDNA synthesis. The first and second strands cDNA synthesis reactions were carried out using TruSeq Stranded Total RNA Sample Prep kit (Illumina, CA, US) according to the manufacturer’s recommendations. Following the first and second cDNA strands synthesis, the samples were end-repaired and adenylated, then Illumina TruSeq adapters (CA, US) were ligated. After adapter ligation, the fragments were enriched using PCR and the products were quantified using Qubit Fluorometer (Life Technologies, NY, US) and Agilent 2100 Bioanalyzer. The libraries were bar-coded using standard Illumina adaptors, purified and size-selected with Agencort AMPure XP beads (Beckman Coulter, CA, US). The quantity and quality of the final sample libraries were verified using a NanoDrop spectrophotometer (ThermoFisher Scientific, DE, US) and 2100 Bioanalyzer (Agilent, CA, US).

**Table 2:** Information on partial segment S1 sequenced from fish samples collected in Alaska, British Columbia and Washington State. Ten types of identical sequences have been identified and grouped in five clusters.

Clusters	Types	GenBank ID	Name	Host species (common name)	Collection Date	Tissue	Location (State, Country)
Cluster 1 (C1)	BCJ31915	KR558677	BC131_13	Farmed *Salmo salar* (Atlantic salmon)	May-13	Heart	DFO area 12 (British Columbia, Canada)
		KR781117	BC1310_13	Farmed *Salmo salar* (Atlantic salmon)	May-13	Heart	DFO area 12 (British Columbia, Canada)
		KR781118	BC1311_13	Farmed *Salmo salar* (Atlantic salmon)	May-13	Heart	DFO area 12 (British Columbia, Canada)
		KR558678	BC132_13	Farmed *Salmo salar* (Atlantic salmon)	May-13	Heart	DFO area 12 (British Columbia, Canada)
		KR558679	BC133_13	Farmed *Salmo salar* (Atlantic salmon)	May-13	Heart	DFO area 12 (British Columbia, Canada)
		KR558680	BC134_13	Farmed *Salmo salar* (Atlantic salmon)	May-13	Heart	DFO area 12 (British Columbia, Canada)
		KR558681	BC135_13	Farmed *Salmo salar* (Atlantic salmon)	May-13	Heart	DFO area 12 (British Columbia, Canada)
		KR558682	BC136_13	Farmed *Salmo salar* (Atlantic salmon)	May-13	Heart	DFO area 12 (British Columbia, Canada)
		KR558683	BC137_13	Farmed *Salmo salar* (Atlantic salmon)	May-13	Heart	DFO area 12 (British Columbia, Canada)
		KR558684	BC138_13	Farmed *Salmo salar* (Atlantic salmon)	May-13	Heart	DFO area 12 (British Columbia, Canada)
		KR558685	BC139_13	Farmed *Salmo salar* (Atlantic salmon)	May-13	Heart	DFO area 12 (British Columbia, Canada)
		KR872637	BC361_14	Farmed *Salmo salar* (Atlantic salmon)	May-14	Heart	Hatchery (British Columbia, Canada)
			BC362_14	Farmed *Salmo salar* (Atlantic salmon)	May-14	Heart	Hatchery (British Columbia, Canada)
			BC363_14	Farmed *Salmo salar* (Atlantic salmon)	May-14	Heart	Hatchery (British Columbia, Canada)
			BC364_14	Farmed *Salmo salar* (Atlantic salmon)	May-14	Heart	Hatchery (British Columbia, Canada)
			BC365_14	Farmed *Salmo salar* (Atlantic salmon)	May-14	Heart	Hatchery (British Columbia, Canada)
			BC366_14	Farmed *Salmo salar* (Atlantic salmon)	May-14	Heart	Hatchery (British Columbia, Canada)
			BC367_14	Farmed *Salmo salar* (Atlantic salmon)	May-14	Heart	Hatchery (British Columbia, Canada)
			BC368_14	Farmed *Salmo salar* (Atlantic salmon)	May-14	Heart	Hatchery (British Columbia, Canada)
		KR347084	BCJ24201_13	Farmed *Salmo salar* (Atlantic salmon)	Sep-13	Heart	DFO area 12 (British Columbia, Canada)
		KR347085	BCJ28529_13	Farmed *Salmo salar* (Atlantic salmon)	Apr-13	Heart	DFO area 7 (British Columbia, Canada)
		KR347086	BCJ28537_13	Farmed *Salmo salar* (Atlantic salmon)	Apr-13	Heart	DFO area 7 (British Columbia, Canada)
		KR347087	BCJ28545_13	Farmed *Salmo salar* (Atlantic salmon)	Apr-13	Heart	DFO area 7 (British Columbia, Canada)
		KR347088	BCJ31910_13	Farmed *Salmo salar* (Atlantic salmon)	Oct-13	Heart	Hatchery (British Columbia, Canada)
		KR347089	BCJ31914_13	Farmed *Salmo salar* (Atlantic salmon)	Oct-13	Heart	Hatchery (British Columbia, Canada)
		KR347090	BCJ31915_13	Farmed *Salmo salar* (Atlantic salmon)	Oct-13	Heart	Hatchery (British Columbia, Canada)
		KR347091	BCJ31916_13	Farmed *Salmo salar* (Atlantic salmon)	Oct-13	Heart	Hatchery (British Columbia, Canada)
		KR347092	BCJ31920_13	Farmed *Salmo salar* (Atlantic salmon)	Oct-13	Heart	Hatchery (British Columbia, Canada)
		KR347094	BCJ35240_13	Farmed *Salmo salar* (Atlantic salmon)	Nov-13	Heart	Hatchery (British Columbia, Canada)
		KR347096	BCJ35249_13	Farmed *Salmo salar* (Atlantic salmon)	Nov-13	Heart	Hatchery (British Columbia, Canada)
		KR347098	BCJ35256_13	Farmed *Salmo salar* (Atlantic salmon)	Nov-13	Heart	Hatchery (British Columbia, Canada)
		KR347095	BCJ35246_13	Farmed *Salmo salar* (Atlantic salmon)	Nov-13	Heart	Hatchery (British Columbia, Canada)
		KR347097	BCJ35255_13	Farmed *Salmo salar* (Atlantic salmon)	Nov-13	Heart	Hatchery (British Columbia, Canada)
		KR347100	BCJ40723_13	Farmed *Salmo salar* (Atlantic salmon)	Nov-13	Heart	Hatchery (British Columbia, Canada)
		KR347102	BCJ40740_13	Farmed *Salmo salar* (Atlantic salmon)	Nov-13	Heart	Hatchery (British Columbia, Canada)
		KR347101	BCJ40731_13	Farmed *Salmo salar* (Atlantic salmon)	Nov-13	Heart	Hatchery (British Columbia, Canada)
		KR347105	BCJ402256_13	Wild *Oncorhynchus kisutch* (Coho salmon)	Nov-13	Heart	Quinsam Hatchery (British Columbia, Canada)
		KR347112	BCK14114_14	Farmed *Salmo salar* (Atlantic salmon)	Apr-14	Heart	DFO area 27 (British Columbia, Canada)
		KR347113	BCK14120_14	Farmed *Salmo salar* (Atlantic salmon)	Apr-14	Heart	DFO area 27 (British Columbia, Canada)
	BCJ402276	KR347106	BCJ402276_13	Wild *Oncorhynchus kisutch* (Coho salmon)	Nov-13	Heart	Quinsam Hatchery (British Columbia, Canada)
Cluster 2 (C2)	BCJ18824	KR347081	BCJ18824_13	Wild *Oncorhynchus tshawytscha* (Chinook salmon)	Aug-13	Heart	DFO area 127 (British Columbia, Canada)
		KR347083	BCJ19943_13	Wild *Oncorhynchus kisutch* (Coho salmon)	Aug-13	Heart	DFO area 127 (British Columbia, Canada)
		KR347093	BCJ34056_13	Wild *Oncorhynchus kisutch* (Coho salmon)	Oct-13	Heart	Quinsam Hatchery (British Columbia, Canada)
		KR347103	BCJ378151_13	Wild *Oncorhynchus kisutch* (Coho salmon)	Nov-13	Heart	Quinsam Hatchery (British Columbia, Canada)
Cluster 3 (C3)	BCJ19323	KR347082	BCJ19323_13	Wild *Oncorhynchus kisutch* (Coho salmon)	Aug-13	Heart	DFO area 7 (British Columbia, Canada)
		KR347104	BCJ378241_13	Wild *Oncorhynchus kisutch* (Coho salmon)	Nov-13	Heart	Quinsam Hatchery (British Columbia, Canada)
		KR347099	BCJ37896_13	Wild *Oncorhynchus kisutch* (Coho salmon)	Nov-13	Heart	Quinsam Hatchery (British Columbia, Canada)
		KR347110	BCK1562_14	Wild *Oncorhynchus kisutch* (Coho salmon)	May-14	Heart	Quinsam Hatchery (British Columbia, Canada)
		KR347115	BCK15625_14	Wild *Oncorhynchus kisutch* (Coho salmon)	May-14	Heart	Quinsam Hatchery (British Columbia, Canada)
		KR347111	BCK1566_14	Wild *Oncorhynchus kisutch* (Coho salmon)	May-14	Heart	Quinsam Hatchery (British Columbia, Canada)
		KR478634	WS1209_12	Wild *Oncorhynchus tshawytscha* (Chinook salmon)	Sep-12	Pool of gill, heart and kidney	Columbia River (Washington State, US)
		KR478637	WSKFH11_14	Wild *Oncorhynchus kisutch* (Coho salmon)	Mar-14	Blood	Columbia River (Washington State, US)
		KR478639	WSKFH13_14	Wild *Oncorhynchus kisutch* (Coho salmon)	Mar-14	Blood	Columbia River (Washington State, US)
		KR478636	WSKFH2_14	Wild *Oncorhynchus kisutch* (Coho salmon)	Mar-14	Blood	Columbia River (Washington State, US)
	WS1207	KR478633	WS1207_12	Wild *Oncorhynchus tshawytscha* (Chinook salmon)	Sep-12	Pool of gill, heart and kidney	Columbia River (Washington State, US)
Cluster 4 (C4)	BCA1338	KR478642	BCA1338_01	Wild *Oncorhynchus tshawytscha* (Chinook salmon)	May-01	Multiple Organs	DFO Area 13 (British Columbia, Canada)
		KR478643	BCA1846_01	Farmed *Salmo salar* (Atlantic salmon)	Aug-01	Multiple Organs	DFO Area 18 (British Columbia, Canada)
		KR478644	BCA1848_01	Farmed *Salmo salar* (Atlantic salmon)	Aug-01	Multiple Organs	DFO Area 18 (British Columbia, Canada)
		KR347078	BCA1849_01	Farmed *Salmo salar* (Atlantic salmon)	Aug-01	Multiple Organs	DFO Area 18 (British Columbia, Canada)
		KR347079	BCA1850_01	Farmed *Salmo salar* (Atlantic salmon)	Aug-01	Multiple Organs	DFO Area 18 (British Columbia, Canada)
		KR347080	BCA1854_05	Farmed *Salmo salar* (Atlantic salmon)	Mar-05	Head kidney, trunk kidney, liver and spleen	DFO Area 18 (British Columbia, Canada)
		KR347107	BCJ402334_13	Wild *Oncorhynchus kisutch* (Coho salmon)	Nov-13	Heart	Quinsam Hatchery (British Columbia, Canada)
		KR347109	BCK1436_14	Salmo salar (Atlantic salmon)	Apr-14	Heart	DFO area 12 (British Columbia, Canada)
Cluster 5 (C5)	AKJ20115	KR478640	AKJ20115_13	Wild *Oncorhynchus kisutch* (Coho salmon)	Aug-13	Heart	Copper River (Alaska, US)
		KR478641	AKJ20120_13	Wild *Oncorhynchus kisutch* (Coho salmon)	Aug-13	Heart	Copper River (Alaska, US)
		KR872635	BCINOC3_13	Wild *Oncorhynchus tshawytscha* (Chinook salmon)	May-13	Kidney, liver mixture	DFO area 124 (British Columbia, Canada)
		KR478635	WSKFH1_14	Wild *Oncorhynchus kisutch* (Coho salmon)	Mar-14	Blood	Columbia River (Washington State, US)
		KR478638	WSKFH12_14	Wild *Oncorhynchus kisutch* (Coho salmon)	Mar-14	Blood	Columbia River (Washington State, US)
	BCK1435	KR347108	BCK1435_14	Farmed *Salmo salar* (Atlantic salmon)	Apr-14	Heart	DFO area 12 (British Columbia, Canada)
	BCK14310	KR347114	BCK14310_14	Farmed *Salmo salar* (Atlantic salmon)	Apr-14	Heart	DFO area 12 (British Columbia, Canada)
	BCINO12	KR872636	BCINOC12_13	Wild *Oncorhynchus tshawytscha* (Chinook salmon)	May-13	Kidney, liver mixture	DFO area 124 (British Columbia, Canada)

In order to generate clusters of reads, the run library was loaded at 10 pmol onto Illumina MiSeq^™^ System (Illumina, CA, US) and paired-end 2 x 300 bp sequencing was performed on sample ID# BCJ31915_13. For samples ID# BCJ19943_14 and WSKFH12_14, the run library mix was loaded at 1.5 pmol into NextSeq 500^™^ (Illumina, CA, US); paired-end 2 x 150 bp sequencing was performed on both samples.

### PRV Genome Sequence Assembly

Sequence assembly was performed using CLC Genomic Workbench (v6.0). Adaptors and low quality bases were discarded and sequence reads were aligned to the Canadian PRV reference genome generated from sample VT06062012-358 [10]. Mapping parameters are mismatch cost = 2, insertion cost = 3, deletion cost = 3, Length fraction = 0.7 and similarity fraction = 0.85. Consensus sequences were generated using the default parameters apart from the threshold which was set at 20. Consensus sequences were aligned against PRV genome sequences available in GenBank [4, 10].

### Phylogenetic analysis

Piscine reovirus segment S1 was chosen for phylogenetic analysis as other studies have demonstrated its suitability for PRV sequence typing [10, 12]. The detailed information on the PRV S1 sequences used in this analysis is described in Table 2. In order to avoid inserting gaps, an alignment of the nucleotide acids was generated from the alignment of the corresponding amino acids using DAMBE program [13, 14]. Phylogenetic analyses were performed on trimmed, partial PRV genome sequences using both PAUP* (version 4.0 b10) for parsimony analysis and Mega (version 6) for Maximum Likelihood method based on the Kimura 2-parameter model with 1,000 bootstrap replicates [15]. This approach was also used to reconstruct the phylogenetic history of the other 10 PRV genome segments on the three samples selected for genome sequences.

## Results

### Piscine reovirus partial segment S1 sequence analysis

In our previous study, six salmonid species were tested for the presence of PRV RNA in tissue by real time RT-PCR: Atlantic salmon (*Salmo salar*), Coho salmon (*Oncorhynchus kisutch*), Chum salmon, Chinook salmon (*Oncorhynchus tshawytscha*), Sockeye salmon (*Oncorhynchus nerka*) and Pink salmon (*Oncorhynchus gorbuscha*) [11]. PRV RNA was detected in samples derived from Atlantic, Coho and Chinook salmon samples. We also observed positive PRV test results in a subset of Chum salmon samples. However, the Ct values in the positive Chum salmon samples were higher than 30 and could not be used in this phylogenetic study. In our earlier study, we also obtained amplification with high Ct values in a few Sockeye salmon samples but these results could not be confirmed. No Pink salmon samples tested positive for PRV RNA [11].

**Fig 2:**
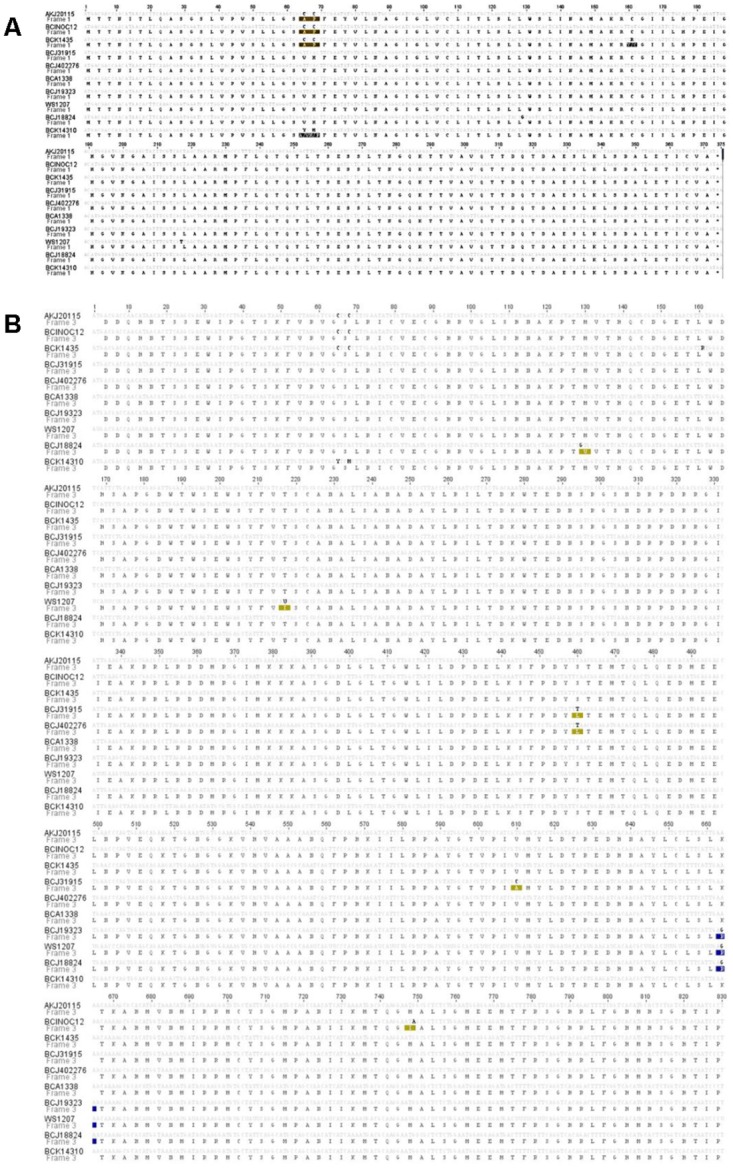
Alignment of the 10 different types of Piscine reovirus partial segment S1 and ORF p13 nucleic acid and amino acid sequences derived from fish tissues collected in the Canada/US Pacific area (see Table 1 for sample identification). Ten distinct sequence types were identified from 71 fish samples. A/ Nucleic acid sequences encoding for protein p13. B/ Partial segment S1 nucleic acid sequences encoding for protein σ3. Grey codes represent conserved nucleotides whereas black letters indicate nucleotide substitutions. Amino acid substitutions are highlighted.

The PRV segment S1 was partially sequenced (830 out of 1,081 total bp) from 71 fish collected from Canada/US Pacific coast between 2001 and 2014 (Table 2). Sequences were analyzed for insertions and deletions using the DAMBE program and no indels were detected. Nucleic acid alignment indicated ten different sequence types that grouped into five general clusters (Fig 2). All samples had a single PRV sequence type except for sample BCK14310_14 (acc# KR347114) which had a mix of two sequence types with ambiguous nucleotides at positions 65 and 68 and BCK1435_14 (acc# KR347108) had an ambiguous nucleotide at position 161.

Sequences showing 100% of nucleotide identity were grouped into 10 different types (Table 2 and Fig 2). Cluster 1 (C1) was represented by PRV S1 sequence types BCJ31915 and BCJ402276. This sequence type was detected commonly in hatchery Atlantic salmon and in wild brood stock Coho salmon collected from the Quinsam Hatchery (B.C.) in November 2013. Cluster 2 (C2) and 3 (C3) were represented by wild Coho and Chinook salmon. The C2 representative sequence type (BCJ18824) was obtained from wild Coho salmon collected in 2013 from DFO area 127 and from Quinsam Hatchery as well as Chinook salmon collected in August 2013 from DFO area 127. Representative C3 sequences (sequence types WS1207 and BCJ19323) were obtained from wild Coho and Chinook salmon sampled in 2012 and 2014 from Columbia River (Washington State, US), as well as Coho salmon from waters of DFO area 7 and Quinsam Hatchery in 2013 and 2014. Cluster 4 (C4) was represented as sequence type BCA1338 and was mainly obtained from archived Atlantic and Chinook salmon tissue samples collected from DFO area 13 and 18 in 2001 and 2005, wild Coho salmon from Quinsam Hatchery sampled in 2013 and saltwater farmed Atlantic salmon from DFO area 12. Cluster 5 (C5) was represented by sequence type AKJ20115, which was found in samples from wild Coho salmon collected from the Copper River in Alaska, by Coho salmon collected from the Columbia River (Washington State, US). Cluster 5 also grouped several unique sequence types from farmed Atlantic salmon sampled from DFO area 12 (sequence types BCK14310 and BCK1435) and wild Chinook salmon from the West coast of Vancouver Island at DFO area 124 (sequence type BCINOC12).

### Piscine reovirus partial segment S1 phylogenetic analysis

**Fig 3:**
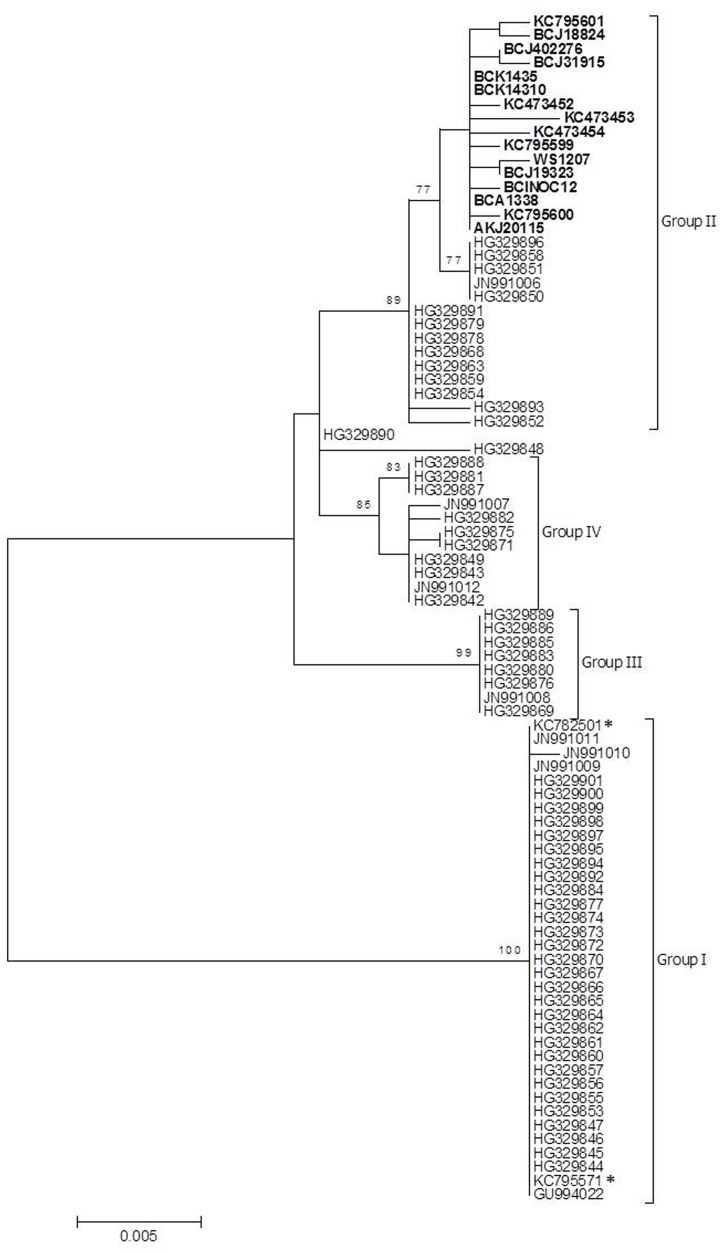
Molecular phylogenetic analysis of unique partial sequences of Piscine reovirus segment S1 derived from fish in the North American Pacific region, Chile and Norway. A total of 830 out of 1,081 nucleotides were used in the analysis. Phylogenetic relationships were inferred using the maximum likelihood method based on the Kimura 2-parameter model. Bootstrap analysis (1,000 replicates) was used to validate tree topology. The numbers above the branches represent the percentage of trees in which the taxa are clustered; only nodes with ≥ 70% bootstrap support are shown. The North American Pacific samples are designated with bold, the Chilean sequences are designated with a star, and the Norwegian sequences are not designated. Sequences derived from this study include BCJ18824, BCJ402276, BCJ31915, BCK1435, BCK14310, WS1207, BCJ19323, BCINOC12, BCA1338, AKJ20115 (See Table 2 for additional sample identification, samples from Norway and Chile are described in S1 Table).

The 71 samples resulted in 10 unique sequence types (Table 2). These 10 sequence types and publically available GenBank sequences were subjected to phylogenetic analysis using the parsimony and maximum likelihood methods. Similar tree topologies were obtained from both methods and results supported four clusters with high bootstrap values (Fig 3 and S1 Table). All North American PRV S1 sequence types clustered with some but not all Norwegian sequence types in Group II. However, the North American sequence types formed a subgroup within Group II with high bootstrap value (Fig 3). The maximum number of nucleotide differences between Norwegian and western North American sequences in Group II were 9 out of 830 bp (1.1%). The maximum nucleotide difference among all the sequences available in GenBank used in the study and western North American sequence types was 38 out of 830 bp (4.6%) which is represented by differences between Group I and western North American sequences (S1 Table). There was no geographical distribution pattern evident for the North American Pacific region. For example, the same partial S1 PRV sequence type (AKJ20115; Table 2) was found in samples from the Copper River in Alaska, DFO area 12 and 124 in British Columbia and the Columbia River in Washington State.

The present study included fish samples freshly collected in 2013 and 2014 from different sites of the North American Pacific region, as well as archived formalin-fixed, paraffin-embedded (FFPE) samples from 2001 and 2005. Based on the S1 sequence analysis and phylogenetic inferences, there was no clear temporal distribution of the different sequences. For example, PRV sequences obtained from archived FFPE sections of Atlantic salmon collected in 2001 and 2005 have the same partial S1 segment sequences as wild Coho salmon collected in 2013 from Quinsam Hatchery as well as farmed Atlantic salmon sampled in 2014 from DFO Area 12 (sequence type BCA1338; Table 2).

### Protein p13 open reading frame sequence analysis

**Fig 4:**
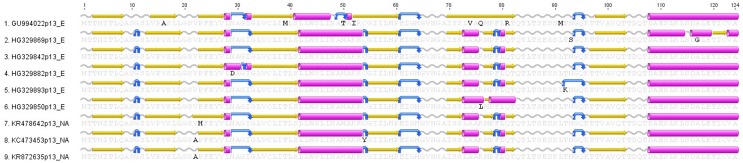
Amino acid alignment of open reading frame consensus sequences encoding the Piscine reovirus p13 protein. Secondary structure and transmembrane domains were predicted using EMBOSS 6.6.7 (Geneious software v6.1). Predicted secondary structure of alpha helix, beta strand, coil and turn are presented in purple cylinders, yellow arrows, grey sinusoids and blue curved arrow. Detailed information of individual sequences are presented in S2 Table.

The PRV S1 segment has an internal open reading frame (ORF) that encodes the p13 protein, which is hypothesized to play a key role in the cytotoxicity of the virus [9]. Amino acids alignment was performed on the 143 sequences listed in S2 Table. Nine consensus sequences have been identified including six from Norway (GU994022p13_E, HG329869p13_E, HG329842p13_E, HG329882p13_E, HG329893p13_E, HG329850p13_E) and three from the Canada/US Pacific coast (KR478642p13_NA, KC473453p13_NA, KR872635p13_NA). Interestingly, the Norwegian consensus GU994022p13_E, HG329842p13_E and HG329850p13_E include sequences from fish with HSMI and with no lesions (S2 Table). Alignment of consensus sequences showed that Norwegian GU994022p13_E has 8 amino acids variants in comparison to the other consensus sequences, while identified nucleic acid variants are silent and have no frame shift effect on the proteins (Fig 4). However, the secondary structure of p13 amino acid sequences predicted using EMBOSS 6.6.7 [16] implemented by the Geneious software (version 6.1.) showed some differences.

### Piscine reovirus partial genome analysis

Piscine reovirus RNA identified from hatchery Atlantic salmon collected in B.C. (ID# BCJ31915_13), wild Coho salmon from DFO Area 7 (ID# BCJ19943_13) and Coho salmon from the Columbia River (ID# WSKFH12_14) were sequenced using next generation of sequencing (Illumina platform). Summary of generated reads is reported in S3 Table. Briefly, 367,092,107; 22,011,189 and 90,260,030 reads were generated for BCJ19943_13, BCJ31915_13 and WSKFH12_14 respectively. Reads with low quality (Q < 20) and length lower than 20 bp were removed prior to further analysis.

Sequences from a Canadian sample VT06062012-358 [10] was used as reference to map the reads. Consensus sequences were generated and aligned against sequences of each segment from a Norwegian representative [4], a Chilean representative [10] and two Canadian ones [10]. In order to verify the quality of next generation sequence analysis, consensus sequences of S1 segment were aligned against S1 segment partial sequences generated by Sanger sequencing and used for the phylogenetic tree analysis. Segment S1 sequences generated from next generation of sequencing matched 100% the sequences generated by Sanger sequencing.

Due to low sequence quality of the genome segment ends, only partial PRV sequences of the 10 segments have been analysed (S3 Table). Between 95.7% and 97.9% of whole PRV sequences were generated in this study. The percentage of variants ranged between 0% and 3.7%. The highest percentage of nucleic acid variants is recorded for segments M2 and S1 from Norwegian [4] and Chilean [10] sequences which were clustered within Group I (Fig 3).

**Fig 5:**
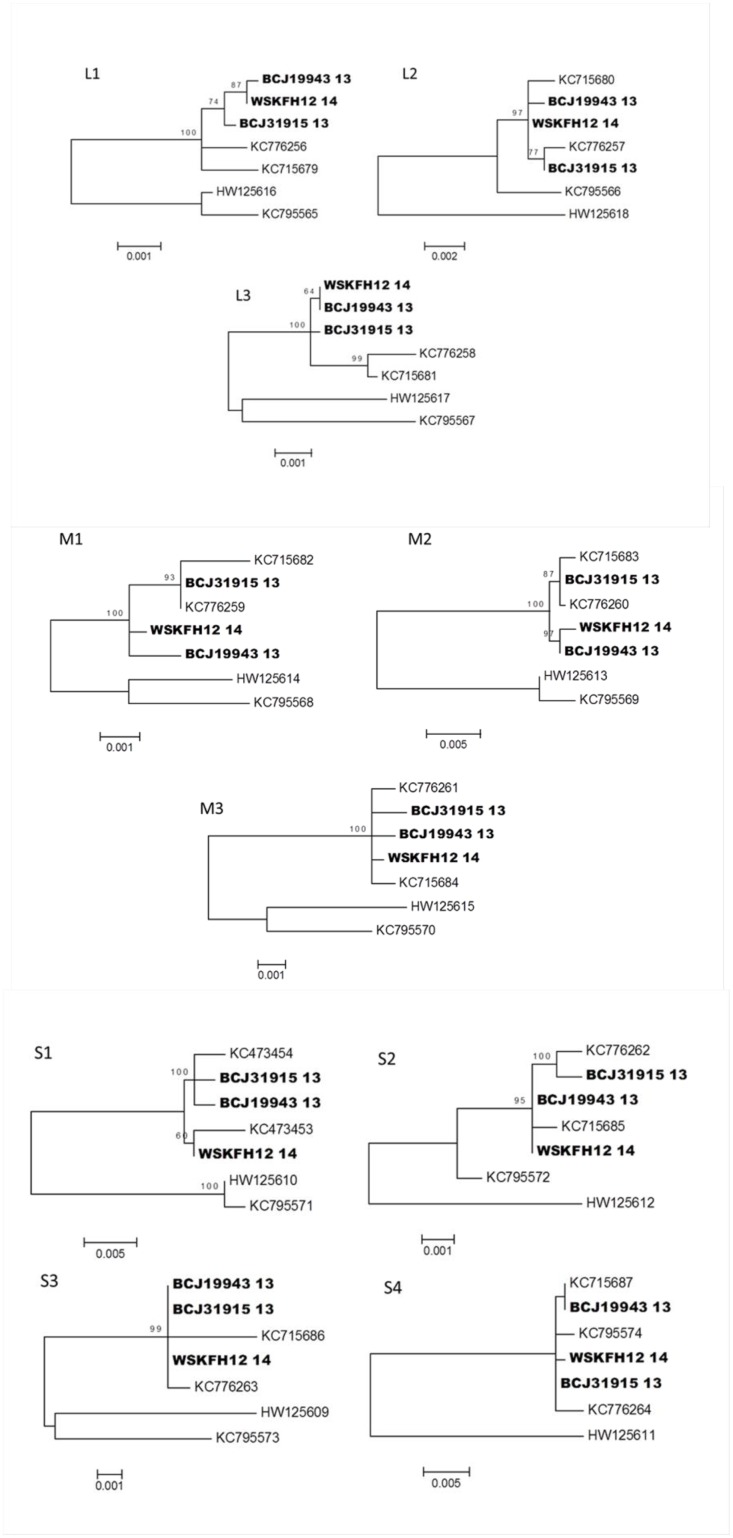
Phylogenetic relationships of Piscine reovirus sequence types derived from North American Pacific samples, a representative Norwegian and a representative Chilean sample. Samples BCJ31915_13, BCJ19943_13 and WSKFH12_14 (see Table 1 for sample identification) were sequenced using the Illumina platform and sequences from all 10 genome segments (L1, L2 L3, M1, M2, M3, S1, S2, S3, S4) were obtained. Sequences derived from this study are designated with bold.

Phylogenetic analysis using maximum likelihood with 1,000 bootstrap replicates was performed on each segment (Fig 5). Analysis of all segments except S4 grouped all North American samples with each other, while the Norwegian and Chilean representative samples formed a separate group. In contrast, the segment S4 sequence analysis did not resolve the Norwegian, Chilean and North American samples (Fig 5). For segment S4, no bootstrap values higher than 70% were obtained due to low variability between the different sequences.

### PRv S1 and M2 open reading frame sequence analysis

**Fig 6:**
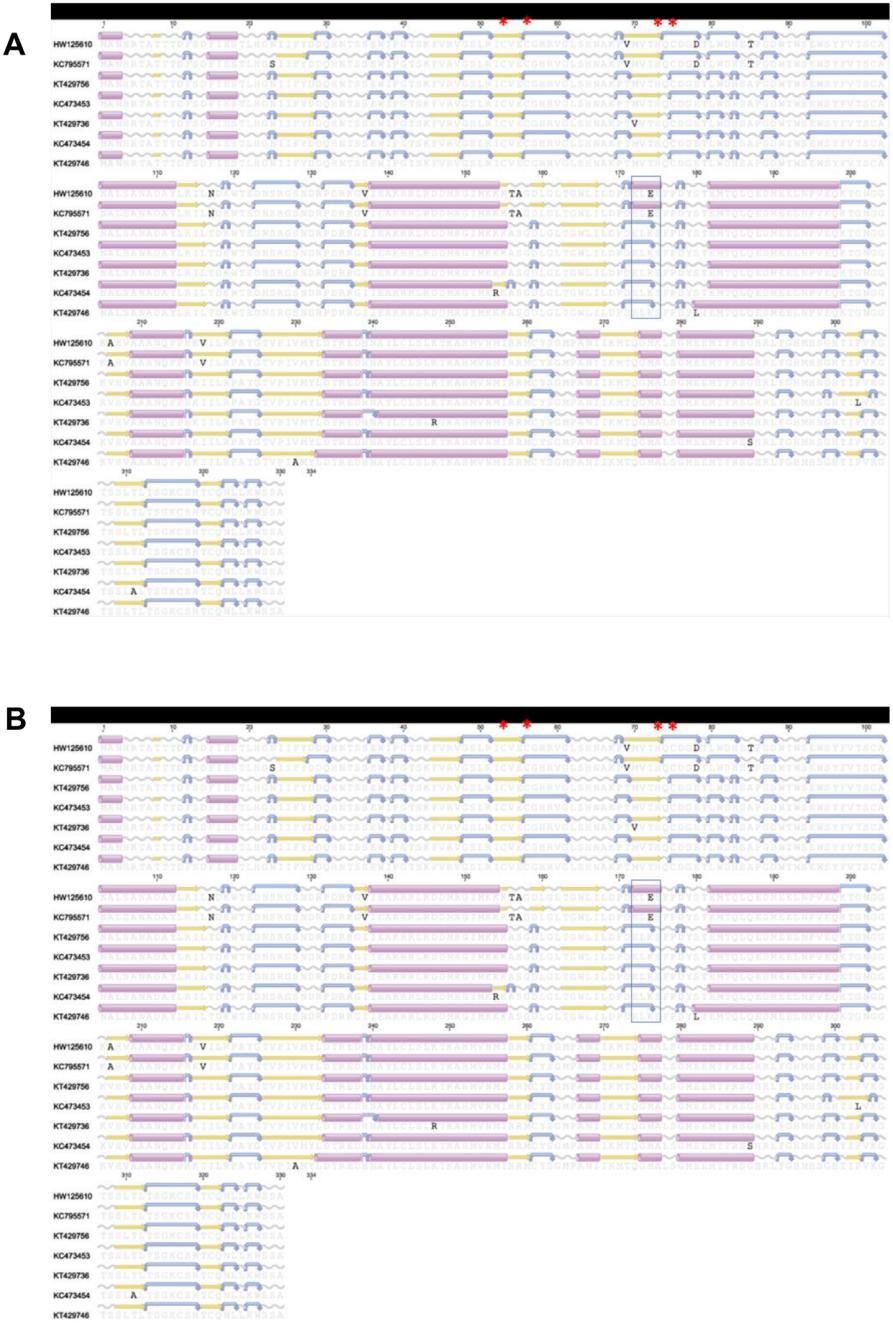
Amino acid alignment of open reading frame consensus sequences encoding the Piscine reovirus σ3 and μ1 protein. Secondary structure and transmembrane domains were predicted using EMBOSS 6.6.7 (Geneious software v6.1). Predicted secondary structure of alpha helix, beta strand, coil and turn are presented in purple cylinders, yellow arrows, grey sinusoids and blue curved arrow. Sequences are identified using the GenBank accession numbers. A/ represents ORF sequences encoding PRV σ3 amino acid alignment. Red stars are conserved Zn-finger motifs. B/ represents ORF sequences encoding PRV μ1 amino acid alignment. Red cross is myristoylation site in the MRV protein and green line is post-translational cleavage site in MRV and ARV [7].

The PRV S1 and M2 segments have open reading frame (ORF) that encode the major outer capsid σ3 and μ1 proteins, respectively. These proteins play key roles in membrane penetration and infectivity of mammalian reoviruses [7]. Amino acids alignment showed high similarities of sequences between the different sequences for both ORF encoding proteins (Fig 6). Zn-finger motifs of σ3 protein are conserved among the different sequences (Fig 6A) as well as myristoylation and post-translational cleavage sites described in mammalian and avian reoviruses (Fig 6B). However, our alignment highlighted some amino acid differences between PRV sequences from Norway (HW125610)/Chile (KC795571) and West coast of North America. For example, at a position 174 of σ3 protein sequences, glutamic acid (E) in Norwegian/Chilean is substituted by lysine (K) in Canadian/US. This substitution leads to a secondary structure modification from “alpha helix” structure in Norwegian/Chilean to a “turn” structure in Canadian/US sequences (Fig 6A). Alignment of μ1 amino acids showed a substitution of serine (S) from Norwegian/Chilean to alanine (A) from Canadian/US at position 262 which leads to a shift from β strand in Norwegian/Chilean structure to α helix structure in Canadian/US sequences (Fig 6B).

## Discussion

The goal of the present study was to investigate phylogenetic relationships among PRV sequence types present in western North America. The primary target for PRV phylogenetic analysis used by ourselves and others has been the bicistronic S1 segment which encodes the major outer capsid protein σ3 and a non-fusogenic, cytotoxic protein identified as p13 [7, 9]. The PRV σ3 outer capsid protein also binds dsRNA; the mammalian orthoreovirus σ3 protein binds dsRNA in a similar fashion and functions to inhibit innate immune responses [17]. A previous study examining PRV segment S1 sequence variation in British Columbia (B.C.) salmon and trout reported low levels of nucleotide variation within B.C. (1%), as well as low levels of divergence between B.C. sequence types and some sequence types reported from Norway [10]. However, this previous study evaluated a limited number of samples and the precise geographic location and origin of the fish samples used in the study were not clearly defined. In the present study, we expanded the geographic range of samples surveyed to include Alaska (US), B.C. (Canada) and Washington State (US). Our overall results were consistent with the previous study and indicated high genetic homogeneity within western North America and high similarity to some Norwegian sequence types that cluster within Group II (Fig 3). It is noteworthy that the Norwegian sequences including the two Chilean sequences (KC782501 and KC795571) of Group I differ by more than 4% with Group II sequences. This indicates a high variability of PRV segment S1 in the Norwegian area. In contrast, the western North American S1 sequences suggest a low level of PRV diversity in this area (Fig 3 and S1 Table).

The reason why there is a low level of nucleotide divergence observed among the PRV samples sequenced to date is not clear but one hypothesis could be that PRV was only recently introduced into North America and/or Europe. Viruses introduced into a new environment typically have high genetic homogeneity early after introduction, but evolutionary divergence can occur rapidly in a new environment. For example, genetic typing of the highly pathogenic fish virus viral hemorrhagic septicemia virus (VHSV; strain IVb) in the first 7 years following its discovery in the North American Great Lakes indicated low genetic diversity (maximum nucleotide diversity 1%) of isolates [18]. More recent genotyping has indicated increasing diversification of VHSV IVb over time, but still within a low overall level of genetic diversity (Kurath and Tesfaye, pers. comm.). The genetic typing results were consistent with epidemiological data suggesting that VHSV was introduced shortly before the occurrence of massive fish kills. The high genetic homogeneity of PRV S1 sequences in western Canada led Kibenge et al (2013) [10] to conclude that PRV was recently introduced into B.C. waters and dated the divergence from Norwegian sequence types between 2006 and 2008 using molecular clock analysis. However, there is little supporting epidemiological evidence for this hypothesis as there were no documented direct imports of fish eggs into western North America from Norway in that time frame. Additionally, a survey of archived samples by Marty et al (2014) [11] found that PRV RNA was common in fish tissues in B.C. since 1987, and was likely present as early as 1977. The distribution of PRV from Alaska to Washington State further suggests that the virus has been established in western North America for a long enough time to become relatively widespread.

While European origin Atlantic salmon have been introduced to B.C. and Washington State, there has also been extensive transplantation of native Pacific salmon and trout eggs into Europe and elsewhere [19]. Transplanted rainbow trout contributed to the spread of infectious hematopoietic necrosis virus (IHNV), a salmonid virus endemic to western North America, to Europe and Asia [20, 21]. If PRV was endemic to western North America, it is equally probable that movement of infected Pacific salmon or trout eggs could have concomitantly spread PRV in Europe. There have been no published retrospective studies of archived samples conducted in Norway to determine how long the virus has been present in that country. However, Atlantic salmon tissues from Norway collected in 1988 tested positive for PRV RNA (Rimstad pers. comm.) suggesting that the virus was present at least a decade prior to the first reports of HSMI. There has been little surveillance for this virus outside Norway and Western North America. Thus, it is premature to speculate about transmission pathways given the lack of understanding of the global distribution of PRV.

Our characterization of 830 bp of the PRV S1 segment in samples derived from a large geographical range (Alaska to Washington State) and spanning a significant time period (2001–2014) revealed ten distinct sequence types with ≤ 1.1% maximum nucleotide diversity. The homogeneity was exemplified by the AKJ20115 sequence type that was identical in samples from Alaska, B.C. and Washington State and the BCA1338 sequence type that was observed to be identical over a 13-year-period. Genetic and temporal homogeneity is not unique to PRV, as similar patterns have been observed for several other western North American Pacific salmon RNA viruses. The Pacific salmon paramyxovirus (PSPV) is typically isolated from Pacific salmon at spawning but PSPV has never been associated with disease [22]. PSPV isolates group into two major lineages designated A and B based on partial sequencing of 505 nucleotides of the polymerase gene. A comparison of 43 sublineage A isolates collected from Alaska to California over a 24-year-period (1983–2007) revealed only 13 distinct sequence types with 1.4% maximum nucleotide divergence. Isolates obtained from distinct geographic locations (Oregon and California) and separated by over a decade yielded identical sequence types. A similar pattern was observed with sublineage B (0.6% nucleotide maximum nucleotide divergence) but fewer isolates were analyzed. Sequencing of another western North American endemic aquatic rhabdovirus, viral hemorrhagic septicemia virus (VHSV) genotype IVa, revealed 1.5% nucleotide diversity in 669 nucleotides of the glycoprotein (G) gene among isolates collected from Alaska to California over a five-year period [23]. An in-depth study of 63 VHSV IVa isolates specifically derived from B.C. also found low nucleotide diversity (2% maximum nucleotide diversity for 1,524 nucleotides of the G gene) [24]. The IHNV U genogroup is an endemic virus strain type that is widely distributed from Alaska to Oregon and primarily associated with diseases in Sockeye salmon. The sequencing of 303 nucleotides of the IHNV G gene from 180 isolates ranging from Alaska to Oregon over a 20-year period revealed only 3% maximum nucleotide diversity. Thus, the levels of nucleotide diversity we observed for PRV in western North America are certainly at the lower end of the range but not substantially lower than levels observed for other fish RNA viruses in the region.

RNA viruses can undergo rapid evolution because of the exceptionally high mutational rate of RNA polymerases [25]. Maintenance of high levels of genetic homogeneity in an RNA virus over a large geographic or temporal range could be explained by several hypotheses. Low sequence diversity may indicate an established host-pathogen relationship at a relatively stable fitness peak. Additionally, lack of geographical differentiation may reflect mixing of host and virus populations or common environmental reservoir(s) for the virus. Kurath et al (2003) [26] hypothesized that the low evolutionary rate and low frequency of non-synonymous substitutions in the IHNV U genogroup indicates that this genogroup could be at a stable fitness peak in its host, Sockeye salmon. However, it is also known that Pacific salmon species from mid-Oregon to Alaska overlap during the marine phase and this corresponds to the known range of the IHNV U genogroup. In our study, fish collected from the same population and at the same time have sequences from different types and clusters. For instance, Coho salmon collected at the same time and from the same Columbia River population have two distinct sequence types that group with either Cluster C3 or C5 (Table 2). It is noteworthy that Cluster C3 has sequences from fish collected from Quinsam Hatchery in B.C., while Cluster C5 has sequences from fish collected in Alaska and B.C. (Table 2). If PRV transmission occurs readily in the ocean, then the discrete Pacific salmon species and populations essentially constitute one large host population for the virus (as it has been previously suggested for the IHNV U genogroup by [26]). Evidence to date suggests PSPV and IHNV primarily infect salmonids and significant alternative hosts for the virus have not been identified. In contrast, VHSV IVa infects a wide range of marine fish species including Atlantic and Pacific salmon [24]. VHSV IVa can be highly prevalent in forage fish species including Pacific herring (*Cuplea pallasii*) and pilchard (*Sardinops sagax*) and these forage fish may be a consistent and stable reservoir for the virus. Although surveillance for PRV has primarily focused on salmonids, PRV RNA was detected in marine fish from coastal Norway but there was no genetic characterization of the sequence types [27]. No studies to date have tested marine fish from western North America for PRV RNA. In summary, the mechanisms contributing to temporal and geographic genetic homogeneity of PRV are not known but more information is needed about the host—pathogen relationship, as well as prevalence of PRV in non-salmonid species.

Segment S1 was used in the present study and by others to reconstruct the phylogenetic relationships among PRV samples. However, other genome segments could provide additional information on the variability of PRV collected from different species and geographical locations. For that reason, we conducted genome sequencing of several representative sequences of fish from hatchery Atlantic salmon collected in B.C., wild Coho salmon from DFO Area 7, and Coho salmon from the Columbia River. The genome sequences were compared to the Canadian [10], Norwegian [4] and Chilean [10] PRV sequences (S3 Table). Overall, all the segments provided similar phylogenetic trees except for S4 segment (Fig 5). Comparative analysis of S4 segment identified indels that predict a frameshift in some sequences from Norway in comparison to Canadian and Chilean sequences (data not shown). There is a limited number of samples that have been subjected to complete S4 sequencing and the Norwegian sequence types that possess the frame shift were deposited by a single research group [4]. Genome analysis revealed that the highest percentage of nucleotide variability was recorded for segments M2 and S1. Comparative study of reoviruses isolated from mammalian (MRV), grass carp (GCRV) and avian (ARV) species revealed the involvement of S1 and M2 encoded proteins in the membrane penetration and infectivity [7]. Studies on MRV showed that σ3 and μ1 protein form a complex which play a key role in the infection [28]. In our analysis, we did observe some amino acids difference between the sequences from Norway/Chile and Canada/US. For instance, glutamic acid (E) in Norwegian/Chilean is substituted by lysine (K) in Canadian/US at a position 174 of σ3 protein and serine (S) from Norwegian/Chilean is substituted into alanine (A) from Canadian/US at position 262 of μ1 amino acid sequence. These substitutions lead to some secondary structure modifications. However, the main motif playing a key role in the protein functionality such as myristoylation and post-translational sites are conserved among the different sequences.

An infectious etiology for HSMI was demonstrated through an experimental challenge of naïve fish injected with filtered supernatant derived from HSMI diseased fish [2]. A decade later, PRV was identified and hypothesized to be the causal agent of HSMI through the use of 454 pyrosequencing [4]. Several studies show a link between the presence and PRV RNA copy number with the level of HSMI severity in Atlantic salmon. Palacios et al (2010) [4] showed a correlation between PRV load and HSMI outbreak and Finstad et al (2012) [29] identified PRV antigen in leucocyte-like cells and cardiomyocytes of HSMI diseased fish. However, the prevalence of PRV in asymptomatic fish has raised questions as to whether PRV is the sole etiological agent of HSMI. Previous efforts comparing S1 genetic variation in virus sequences obtained from either HSMI-diseased or HSMI-free fish were not conclusive [5]. In our analysis, we did observe amino acid differences in the S1 encoded p13 protein in the North American, Chilean and some Norwegian sequences that altered the predicted secondary structure but no frame shift has been shown in the alignment. In addition, p13 coding sequences from samples of HSMI-diseased fish are 100% similar to sequences from samples of healthy fish. For example, p13 coding sequences derived from experimental HSMI-induced fish (GenBank #GU994022, [4]) are 100% similar to several sequences derived from healthy fish and grouped under GU994022p13_E (S2 Table). Considerably more comparative work is needed to evaluate the hypothesis that HSMI disease is associated with changes in the PRV genome that alter virulence.

In previous study performed by Kibenge et al [10], the authors examined PRV segment S1 sequences variation within British Columbia salmon and trout samples recently collected in 2012. In the present study, we analyzed PRV sequences obtained from samples of wild and farmed salmonids collected across an expanded geographic range from Alaska to Washington State over 13 year period. The phylogenetic analysis of partial PRV S1 sequences from western North America Pacific Region indicated high genetic homogeneity and they form a subgroup within Group II. Little genetic differentiation was observed among sequence types since 2001. This suggests that the circulating virus sequence types are relatively stable in western North American Pacific waters and rules out a recent introduction of PRV into the western North Pacific as suggested by Kibenge et al [10]. However, the mechanisms by which the virus is globally distributed, as well as transmission pathways remain to be elucidated.

## Supporting Information

S1 Fig Molecular phylogenetic analysis of unique partial sequences of Piscine reovirus segment S1 derived from fish in the North American Pacific region, Chile and Norway. A total of 830 out of 1,081 nucleotides were used in the analysis. Phylogenetic relationships were inferred using parsimony method. Bootstrap analysis (1,000 replicates) was used to validate tree topology.
(DOCX)

S1 Table Number and percentage of variants recorded from Piscine reovirus partial S1 segment sequenced fom fish samples collected in Alaska, British Columbia and Washington State in comparison to sequences available in GenBank. (XLSX)

S2 Table Summary of detailed information on p13 encoding sequences used in this study. Nine consensus sequences were generated based on identical sequences. HSMI: Heart and skeletal muscle inflammation, N/A: histology was not performed, NL: No lesion linked to HSMI has been recorded by histology.
(DOCX)

S3 Table Summary of the nucleic comparison between the three PRV genome sequenced in this study from samples (BCJ31915_13, BCJ19943_13 and WSKFH12_14) and the reference sequences. In brackets are represented the sequence size ratio generated in this study and full PRV genome sequences available in GenBank and published by Kibenge et al. (2013) and Palacios et al. (2010). The number and percentage of nucleic acid variants identified from each segment were also recorded.
(XLSX)
