# Correction: Piscine Reovirus: Genomic and Molecular Phylogenetic Analysis from Farmed and Wild Salmonids Collected on the Canada/US Pacific Coast

**DOI:** 10.1371/journal.pone.0164926

**Published:** 2016-10-12

**Authors:** Ahmed Siah, Diane B. Morrison, Elena Fringuelli, Paul Savage, Zina Richmond, Robert Johns, Maureen K. Purcell, Stewart C. Johnson, Sonja M. Saksida

In “Piscine Reovirus: Genomic and Molecular Phylogenetic Analysis from Farmed and Wild Salmonids Collected on the Canada/US Pacific Coast,” by Siah et al. [1], clarifications were needed in regards to the following: (a) the sampling population used in this paper versus that of Kibenge et al. [2] and; (b) the discussion of work by Kibenge et al. [2]. Additionally, there was an error in Fig 6 and Table 2. Here, the authors would like to provide some additional information about the methods used in the PLOS ONE article, clarify the discussion, and correct the aforementioned Figure and Table.

## Comparison of the sampling population used in this article versus that of Kibenge et al. [2]

The authors would like to clarify the differences in the sampling population used in Siah et al. [1] versus Kibenge et al. [2], since such differences may contribute to the different conclusions reached in these articles:

In the present study [1], the samples were selected from extensive PRV surveys performed on the west coast of Canada and the US. Salmonids archived paraffin blocks from 1974 to 2008 (n = 363), fresh-frozen samples from 2013–2014 (n = 1,838) from wild and farmed fish collected in British Columbia, fresh-frozen samples from fish collected in Alaska (n = 295) and fresh or RNA-later preserved samples from fish collected in Washington State (n = 724) were analyzed with real-time RT-PCR [3]. Only samples (n = 71) with Ct values lower than 30 and from which the authors were able to amplify a PCR product were used for this study. Our work extended our knowledge of PRV sequence diversity across a larger geographical range. Additionally, the authors found that partial segment S1 sequence types derived from archived Atlantic and Chinook salmon samples collected in 2001 and 2005 were identical to some PRV sequence types obtained from samples collected in 2013–2014. The phylogenetic analysis of partial PRV S1 sequences from North American Pacific Coast indicated high genetic homogeneity, forming a subgroup within Group II. Little genetic differentiation was observed among sequence types since 2001.

In Kibenge et al. [2], the authors examined PRV segment S1 sequences variation within British Columbia salmon and trout samples (14 samples in total from western Canada) recently collected in 2012.

## Correction to the Discussion, regarding work by Kibenge et al. [2]

In the last paragraph of the current study [1], Siah et al. conclude, "This suggests that the circulating virus sequence types are relatively stable in western North American Pacific waters and rules out a recent introduction of PRV into the western North Pacific as suggested by Kibenge et al [10]." The work by Kibenge et al. was instead done in the eastern north Pacific (off the western coast of Canada), not the western north Pacific. In addition, after careful reconsideration, the authors feel this conclusion is overstated. The authors would like to correct these two issues with the following revision to the final paragraph:

In previous study performed by Kibenge et al [10], the authors examined PRV segment S1 sequences variation within British Columbia salmon and trout samples recently collected in 2012. In the present study, we analyzed PRV sequences obtained from samples of wild and farmed salmonids collected across an expanded geographic range from Alaska to Washington State over 13 year period. The phylogenetic analysis of partial PRV S1 sequences from western North America Pacific Region indicated high genetic homogeneity and they form a subgroup within Group II. In addition, the results presented here suggest that salmonids from western North America Pacific waters carried PRV RNA sequences for at least 13 years with little genetic differentiation among sequence types in selected samples spanning 2001 to 2014. However, the mechanisms by which the virus is globally distributed, as well as transmission pathways remain to be elucidated.

Please see the correct Fig 6 here.

**Fig 6:**
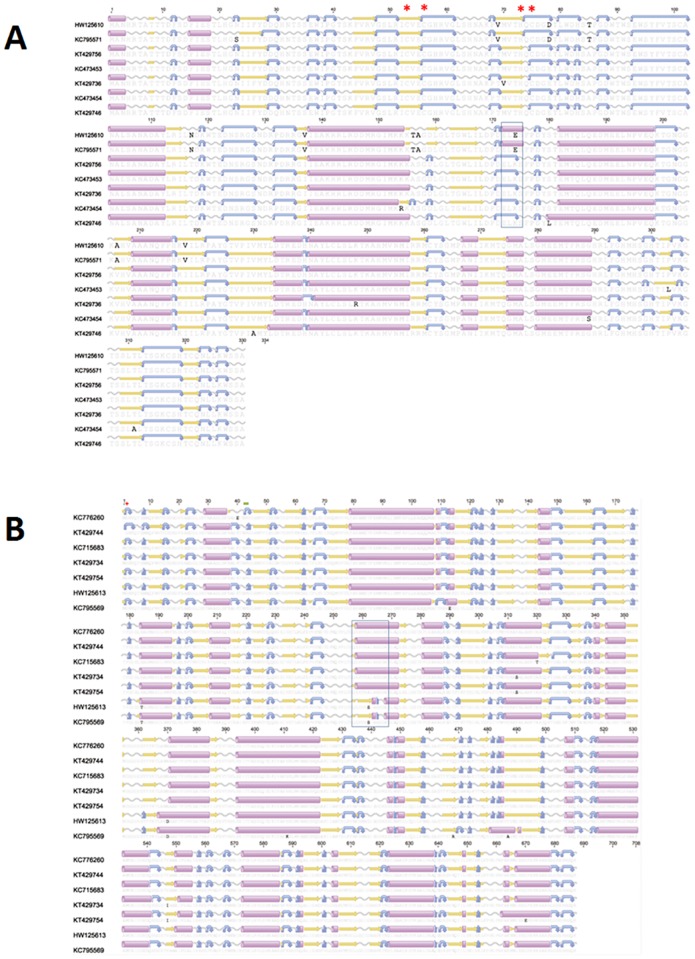
Amino acid alignment of open reading frame consensus sequences encoding the Piscine reovirus σ3 and μ1 protein. Secondary structure and transmembrane domains were predicted using EMBOSS 6.6.7 (Geneious software v6.1). Predicted secondary structure of alpha helix, beta strand, coil and turn are presented in purple cylinders, yellow arrows, grey sinusoids and blue curved arrow. Sequences are identified using the GenBank accession numbers. A/ represents ORF sequences encoding PRV σ3 amino acid alignment. Red stars are conserved Zn-finger motifs. B/ represents ORF sequences encoding PRV μ1 amino acid alignment. Red cross is myristoylation site in the MRV protein and green line is post-translational cleavage site in MRV and ARV [7].

Please see the correct Table 2 here.

**Table 2:** Information on partial segment S1 sequenced from fish samples collected in Alaska, British Columbia and Washington State. Ten types of identical sequences have been identified and grouped in five clusters.

Clusters	Types	GenBank ID	Name	Host species (common name)	Collection Date	Tissue	Location (State, Country)
Cluster 1 (C1)	BCJ31915	KR558677	BC131_13	Farmed *Salmo salar* (Atlantic salmon)	May-13	Heart	DFO area 12 (British Columbia, Canada)
		KR781117	BC1310_13	Farmed *Salmo salar* (Atlantic salmon)	May-13	Heart	DFO area 12 (British Columbia, Canada)
		KR781118	BC1311_13	Farmed *Salmo salar* (Atlantic salmon)	May-13	Heart	DFO area 12 (British Columbia, Canada)
		KR558678	BC132_13	Farmed *Salmo salar* (Atlantic salmon)	May-13	Heart	DFO area 12 (British Columbia, Canada)
		KR558679	BC133_13	Farmed *Salmo salar* (Atlantic salmon)	May-13	Heart	DFO area 12 (British Columbia, Canada)
		KR558680	BC134_13	Farmed *Salmo salar* (Atlantic salmon)	May-13	Heart	DFO area 12 (British Columbia, Canada)
		KR558681	BC135_13	Farmed *Salmo salar* (Atlantic salmon)	May-13	Heart	DFO area 12 (British Columbia, Canada)
		KR558682	BC136_13	Farmed *Salmo salar* (Atlantic salmon)	May-13	Heart	DFO area 12 (British Columbia, Canada)
		KR558683	BC137_13	Farmed *Salmo salar* (Atlantic salmon)	May-13	Heart	DFO area 12 (British Columbia, Canada)
		KR558684	BC138_13	Farmed *Salmo salar* (Atlantic salmon)	May-13	Heart	DFO area 12 (British Columbia, Canada)
		KR558685	BC139_13	Farmed *Salmo salar* (Atlantic salmon)	May-13	Heart	DFO area 12 (British Columbia, Canada)
		KR872637	BC361_14	Farmed *Salmo salar* (Atlantic salmon)	May-14	Heart	Hatchery (British Columbia, Canada)
			BC362_14	Farmed *Salmo salar* (Atlantic salmon)	May-14	Heart	Hatchery (British Columbia, Canada)
			BC363_14	Farmed *Salmo salar* (Atlantic salmon)	May-14	Heart	Hatchery (British Columbia, Canada)
			BC364_14	Farmed *Salmo salar* (Atlantic salmon)	May-14	Heart	Hatchery (British Columbia, Canada)
			BC365_14	Farmed *Salmo salar* (Atlantic salmon)	May-14	Heart	Hatchery (British Columbia, Canada)
			BC366_14	Farmed *Salmo salar* (Atlantic salmon)	May-14	Heart	Hatchery (British Columbia, Canada)
			BC367_14	Farmed *Salmo salar* (Atlantic salmon)	May-14	Heart	Hatchery (British Columbia, Canada)
			BC368_14	Farmed *Salmo salar* (Atlantic salmon)	May-14	Heart	Hatchery (British Columbia, Canada)
		KR347084	BCJ24201_13	Farmed *Salmo salar* (Atlantic salmon)	Sep-13	Heart	DFO area 12 (British Columbia, Canada)
		KR347085	BCJ28529_13	Farmed *Salmo salar* (Atlantic salmon)	Apr-13	Heart	DFO area 7 (British Columbia, Canada)
		KR347086	BCJ28537_13	Farmed *Salmo salar* (Atlantic salmon)	Apr-13	Heart	DFO area 7 (British Columbia, Canada)
		KR347087	BCJ28545_13	Farmed *Salmo salar* (Atlantic salmon)	Apr-13	Heart	DFO area 7 (British Columbia, Canada)
		KR347088	BCJ31910_13	Farmed *Salmo salar* (Atlantic salmon)	Oct-13	Heart	Hatchery (British Columbia, Canada)
		KR347089	BCJ31914_13	Farmed *Salmo salar* (Atlantic salmon)	Oct-13	Heart	Hatchery (British Columbia, Canada)
		KR347090	BCJ31915_13	Farmed *Salmo salar* (Atlantic salmon)	Oct-13	Heart	Hatchery (British Columbia, Canada)
		KR347091	BCJ31916_13	Farmed *Salmo salar* (Atlantic salmon)	Oct-13	Heart	Hatchery (British Columbia, Canada)
		KR347092	BCJ31920_13	Farmed *Salmo salar* (Atlantic salmon)	Oct-13	Heart	Hatchery (British Columbia, Canada)
		KR347094	BCJ35240_13	Farmed *Salmo salar* (Atlantic salmon)	Nov-13	Heart	Hatchery (British Columbia, Canada)
		KR347096	BCJ35249_13	Farmed *Salmo salar* (Atlantic salmon)	Nov-13	Heart	Hatchery (British Columbia, Canada)
		KR347098	BCJ35256_13	Farmed *Salmo salar* (Atlantic salmon)	Nov-13	Heart	Hatchery (British Columbia, Canada)
		KR347095	BCJ35246_13	Farmed *Salmo salar* (Atlantic salmon)	Nov-13	Heart	Hatchery (British Columbia, Canada)
		KR347097	BCJ35255_13	Farmed *Salmo salar* (Atlantic salmon)	Nov-13	Heart	Hatchery (British Columbia, Canada)
		KR347100	BCJ40723_13	Farmed *Salmo salar* (Atlantic salmon)	Nov-13	Heart	Hatchery (British Columbia, Canada)
		KR347102	BCJ40740_13	Farmed *Salmo salar* (Atlantic salmon)	Nov-13	Heart	Hatchery (British Columbia, Canada)
		KR347101	BCJ40731_13	Farmed *Salmo salar* (Atlantic salmon)	Nov-13	Heart	Hatchery (British Columbia, Canada)
		KR347105	BCJ402256_13	Wild *Oncorhynchus kisutch* (Coho salmon)	Nov-13	Heart	Quinsam Hatchery (British Columbia, Canada)
		KR347112	BCK14114_14	Farmed *Salmo salar* (Atlantic salmon)	Apr-14	Heart	DFO area 27 (British Columbia, Canada)
		KR347113	BCK14120_14	Farmed *Salmo salar* (Atlantic salmon)	Apr-14	Heart	DFO area 27 (British Columbia, Canada)
		KR347106	BCJ402276_13	Wild *Oncorhynchus kisutch* (Coho salmon)	Nov-13	Heart	Quinsam Hatchery (British Columbia, Canada)
Cluster 2 (C2)	BCJ18824	KR347081	BCJ18824_13	Wild *Oncorhynchus tshawytscha* (Chinook salmon)	Aug-13	Heart	DFO area 127 (British Columbia, Canada)
		KR347083	BCJ19943_13	Wild *Oncorhynchus kisutch* (Coho salmon)	Aug-13	Heart	DFO area 127 (British Columbia, Canada)
		KR347093	BCJ34056_13	Wild *Oncorhynchus kisutch* (Coho salmon)	Oct-13	Heart	Quinsam Hatchery (British Columbia, Canada)
		KR347103	BCJ378151_13	Wild *Oncorhynchus kisutch* (Coho salmon)	Nov-13	Heart	Quinsam Hatchery (British Columbia, Canada)
Cluster 3 (C3)	BCJ19323	KR347082	BCJ19323_13	Wild *Oncorhynchus kisutch* (Coho salmon)	Aug-13	Heart	DFO area 7 (British Columbia, Canada)
		KR347104	BCJ378241_13	Wild *Oncorhynchus kisutch* (Coho salmon)	Nov-13	Heart	Quinsam Hatchery (British Columbia, Canada)
		KR347099	BCJ37896_13	Wild *Oncorhynchus kisutch* (Coho salmon)	Nov-13	Heart	Quinsam Hatchery (British Columbia, Canada)
		KR347110	BCK1562_14	Wild *Oncorhynchus kisutch* (Coho salmon)	May-14	Heart	Quinsam Hatchery (British Columbia, Canada)
		KR347115	BCK15625_14	Wild *Oncorhynchus kisutch* (Coho salmon)	May-14	Heart	Quinsam Hatchery (British Columbia, Canada)
		KR347111	BCK1566_14	Wild *Oncorhynchus kisutch* (Coho salmon)	May-14	Heart	Quinsam Hatchery (British Columbia, Canada)
		KR478634	WS1209_12	Wild *Oncorhynchus tshawytscha* (Chinook salmon)	Sep-12	Pool of gill, heart and kidney	Columbia River (Washington State, US)
		KR478637	WSKFH11_14	Wild *Oncorhynchus kisutch* (Coho salmon)	Mar-14	Blood	Columbia River (Washington State, US)
		KR478639	WSKFH13_14	Wild *Oncorhynchus kisutch* (Coho salmon)	Mar-14	Blood	Columbia River (Washington State, US)
		KR478636	WSKFH2_14	Wild *Oncorhynchus kisutch* (Coho salmon)	Mar-14	Blood	Columbia River (Washington State, US)
		KR478633	WS1207_12	Wild *Oncorhynchus tshawytscha* (Chinook salmon)	Sep-12	Pool of gill, heart and kidney	Columbia River (Washington State, US)
Cluster 4 (C4)	BCA1338	KR478642	BCA1338_01	Wild *Oncorhynchus tshawytscha* (Chinook salmon)	May-01	Multiple Organs	DFO Area 13 (British Columbia, Canada)
		KR478643	BCA1846_01	Farmed *Salmo salar* (Atlantic salmon)	Aug-01	Multiple Organs	DFO Area 18 (British Columbia, Canada)
		KR478644	BCA1848_01	Farmed *Salmo salar* (Atlantic salmon)	Aug-01	Multiple Organs	DFO Area 18 (British Columbia, Canada)
		KR347078	BCA1849_01	Farmed *Salmo salar* (Atlantic salmon)	Aug-01	Multiple Organs	DFO Area 18 (British Columbia, Canada)
		KR347079	BCA1850_01	Farmed *Salmo salar* (Atlantic salmon)	Aug-01	Multiple Organs	DFO Area 18 (British Columbia, Canada)
		KR347080	BCA1854_05	Farmed *Salmo salar* (Atlantic salmon)	Mar-05	Head kidney, trunk kidney, liver and spleen	DFO Area 18 (British Columbia, Canada)
		KR347107	BCJ402334_13	Wild *Oncorhynchus kisutch* (Coho salmon)	Nov-13	Heart	Quinsam Hatchery (British Columbia, Canada)
		KR347109	BCK1436_14	Salmo salar (Atlantic salmon)	Apr-14	Heart	DFO area 12 (British Columbia, Canada)
Cluster (C5)	AKJ20115	KR478640	AKJ20115_13	Wild *Oncorhynchus kisutch* (Coho salmon)	Aug-13	Heart	Copper River (Alaska, US)
		KR478641	AKJ20120_13	Wild *Oncorhynchus kisutch* (Coho salmon)	Aug-13	Heart	Copper River (Alaska, US)
		KR872635	BCINOC3_13	Wild *Oncorhynchus tshawytscha* (Chinook salmon)	May-13		DFO area 124 (British Columbia, Canada)
		KR478635	WSKFH1_14	Wild *Oncorhynchus kisutch* (Coho salmon)	Mar-14	Blood	Columbia River (Washington State, US)
		KR478638	WSKFH12_14	Wild *Oncorhynchus kisutch* (Coho salmon)	Mar-14	Blood	Columbia River (Washington State, US)
		KR347108	BCK1435_14	Farmed *Salmo salar* (Atlantic salmon)	Apr-14	Heart	DFO area 12 (British Columbia, Canada)
		KR347114	BCK14310_14	Farmed *Salmo salar* (Atlantic salmon)	Apr-14	Heart	DFO area 12 (British Columbia, Canada)
		KR872636	BCINOC12_13	Wild *Oncorhynchus tshawytscha* (Chinook salmon)	May-13		DFO area 124 (British Columbia, Canada)
