# Supplementary material for: Piscine Reovirus: Genomic and Molecular Phylogenetic Analysis from Farmed and Wild Salmonids Collected on the Canada/US Pacific Coast
Source: PLoS One. 2015 Nov 4;10(11):e0141475. doi: 10.1371/journal.pone.0141475 (PMC4633109; doi:10.1371/journal.pone.0141475)
Supplement: S1 Fig — Phylogenetic relationships were inferred using parsimony method. Bootstrap analysis (1,000 replicates) was used to validate tree topology. (DOCX) [file pone.0141475.s001.docx]

69.4

63

68

91.9

100

89

65

70

64

99

66

62

Group I

Group III

Group IV

Group II

Group II
