# Supplementary material for: Piscine Reovirus: Genomic and Molecular Phylogenetic Analysis from Farmed and Wild Salmonids Collected on the Canada/US Pacific Coast
Source: PLoS One. 2015 Nov 4;10(11):e0141475. doi: 10.1371/journal.pone.0141475 (PMC4633109; doi:10.1371/journal.pone.0141475)
Supplement: S2 Table — HSMI: Heart and skeletal muscle inflammation, N/A: histology was not performed, NL: No lesion linked to HSMI has been recorded by histology. (DOCX) [file pone.0141475.s003.docx]

| **Consensus** | **Nucleotide GenBank #** | **Collection Area** | **Collection Year** | **Diagnosis** | **References** |
| --- | --- | --- | --- | --- | --- |
| P13Type1 | GU994022 | experimentally-induced HSMI | N/A | HSMI | Palacios et al. (2010) |
|  | HG329844 | Hatchery reared (Eira, Norway) | 2009 | NL | Garseth et al. (2013) |
|  | HG329845 | Wild (Etne, Norway) | 2009 | NL | Garseth et al. (2013) |
|  | HG329846 | Wild (Etne, Norway) | 2009 | NL | Garseth et al. (2013) |
|  | HG329847 | Wild (Etne, Norway) | 2009 | NL | Garseth et al. (2013) |
|  | HG329853 | Wild (Mandal, Norway) | 2009 | NL | Garseth et al. (2013) |
|  | HG329855 | Escaped farmed (Nidelv, Norway) | 2009 | NL | Garseth et al. (2013) |
|  | HG329856 | Wild (Skibotn, Norway) | 2009 | NL | Garseth et al. (2013) |
|  | HG329857 | Escaped farmed (Skjomen,Norway) | 2009 | NL | Garseth et al. (2013) |
|  | HG329860 | Wild Storelva (Holt, Norway) | 2009 | NL | Garseth et al. (2013) |
|  | HG329861 | Escaped farmed (Surna, Norway) | 2009 | NL | Garseth et al. (2013) |
|  | HG329862 | Escaped farmed Surna (Norway) | 2009 | NL | Garseth et al. (2013) |
|  | HG329864 | Wild (Vosso, Norway) | 2009 | NL | Garseth et al. (2013) |
|  | HG329865 | Hatchery-reared (Vosso, Norway) | 2009 | NL | Garseth et al. (2013) |
|  | HG329866 | Hatchery-reared (Vosso, Norway) | 2009 | NL | Garseth et al. (2013) |
|  | HG329867 | Wild (Alta, Norway) | 2008 | NL | Garseth et al. (2013) |
|  | HG329870 | Wild (Eira, Norway) | 2008 | NL | Garseth et al. (2013) |
|  | HG329872 | Wild(Ekso, Norway) | 2008 | NL | Garseth et al. (2013) |
|  | HG329873 | Wild(Fusta, Norway) | 2008 | NL | Garseth et al. (2013) |
|  | HG329874 | Wild(Gaula, Norway) | 2008 | NL | Garseth et al. (2013) |
|  | HG329877 | Wild(Surna, Norway) | 2008 | NL | Garseth et al. (2013) |
|  | HG329884 | Wild(Eira, Norway) | 2007 | NL | Garseth et al. (2013) |
|  | HG329892 | Wild(Surna, Norway) | 2007 | NL | Garseth et al. (2013) |
|  | HG329894 | Escaped farmed(Halsan, Norway) | 2009 | NL | Garseth et al. (2013) |
|  | HG329895 | Wild(Eidsdal, Norway) | 2008 | NL | Garseth et al. (2013) |
|  | HG329897 | Sea-trout(Moelv, Norway) | 2008 | NL | Garseth et al. (2013) |
|  | HG329898 | Escaped farmed(Etne, Norway) | 2010 | NL | Garseth et al. (2013) |
|  | HG329899 | Escaped farmed(Etne, Norway) | 2010 | NL | Garseth et al. (2013) |
|  | HG329901 | Escaped farmed(Etne, Norway) | 2010 | NL | Garseth et al. (2013) |
|  | HG329900 | Escaped farmed(Etne, Norway) | 2010 | NL | Garseth et al. (2013) |
|  | JN991009 | Norway | 2007 | NL | Løvoll et al. 2012) |
|  | JN991011 | Norway | 2007 | NL | Løvoll et al. 2012) |
|  | JN991010 | Norway | 2007 | HSMI | Løvoll et al. 2012) |
| P13Type2 | HG329869 | Wild(Drevja, Norway) | 2008 | NL | Garseth et al. (2013) |
|  | HG329876 | Wild(Nausta, Norway) | 2008 | NL | Garseth et al. (2013) |
|  | HG329880 | Wild(Vikja, Norway) | 2008 | NL | Garseth et al. (2013) |
|  | HG329883 | Wild(Alta, Norway) | 2007 | NL | Garseth et al. (2013) |
|  | HG329885 | wild(Ekso, Norway) | 2007 | NL | Garseth et al. (2013) |
|  | HG329886 | wild(Ekso, Norway) | 2007 | NL | Garseth et al. (2013) |
|  | HG329889 | Wild(Laerdal, Norway) | 2007 | NL | Garseth et al. (2013) |
|  | JN991008 | Norway | 2007 | NL | Løvoll et al. (2012) |
| P13Type3 | HG329842 | Wild(Bjoreio, Norway) | 2009 | NL | Garseth et al. (2012) |
|  | HG329843 | Hatchery-reared(Eira, Norway) | 2009 | NL | Garseth et al. (2013) |
|  | HG329848 | Wild(Gaula, Norway) | 2009 | NL | Garseth et al. (2013) |
|  | HG329849 | Wild(Hestdal, Norway) | 2009 | NL | Garseth et al. (2013) |
|  | HG329852 | Wild(Mandal, Norway) | 2009 | NL | Garseth et al. (2013) |
|  | HG329854 | Wild(Mandal, Norway) | 2009 | NL | Garseth et al. (2013) |
|  | HG329859 | Wild(Stjordal, Norway) | 2009 | NL | Garseth et al. (2013) |
|  | HG329863 | Wild(Vosso, Norway) | 2009 | NL | Garseth et al. (2013) |
|  | HG329868 | Wild (Alta, Norway) | 2008 | NL | Garseth et al. (2013) |
|  | HG329871 | Wild (Eira, Norway) | 2008 | NL | Garseth et al. (2013) |
|  | HG329875 | Wild( Jolstra, Norway) | 2008 | NL | Garseth et al. (2013) |
|  | HG329878 | Wild (Vikja, Norway) | 2008 | NL | Garseth et al. (2013) |
|  | HG329879 | Wild (Vikja, Norway) | 2008 | NL | Garseth et al. (2013) |
|  | HG329881 | Hatchery-reared (Vosso, Norway) | 2008 | NL | Garseth et al. (2013) |
|  | HG329887 | Wild (Ekso, Norway) | 2007 | NL | Garseth et al. (2013) |
|  | HG329888 | Wild (Ekso, Norway) | 2007 | NL | Garseth et al. (2013) |
|  | HG329890 | Wild (Mandal, Norway) | 2007 | NL | Garseth et al. (2013) |
|  | HG329891 | Wild (Stjordal, Norway) | 2007 | NL | Garseth et al. (2013) |
|  | JN991007 | Norway | 2006 | HSMI | Løvoll et al. (2012) |
|  | JN991012 | Norway | 2006 | HSMI | Løvoll et al. (2012) |
| P13Type4 | HG329882 | Hatchery-reared Vosso, Norway) | 2008 | NL | Garseth et al. (2013) |
| P13Type5 | HG329893 | Wild (Aaroy, Norway) | 2007 | NL | Garseth et al. (2013) |
| P13Type6 | HG329850 | Wild (Hestdal, Norway) | 2009 | NL | Garseth et al. (2013) |
|  | HG329851 | Wild (Hestdal, Norway) | 2009 | NL | Garseth et al. (2013) |
|  | HG329858 | Wild Stjordal (Norway) | 2009 | NL | Garseth et al. (2013) |
|  | HG329896 | Wild Moelv (Norway) | 2008 | NL | Garseth et al. (2013) |
|  | JN991006 | Norway | 2006 | CMS/HSMI | Løvoll et al. (2012) |
| P13Type7 | KR478642 | DFO Area 13 (British Columbia, Canada) | 2001 | NL | This study |
|  | KR478643 | DFO Area 18 (British Columbia, Canada) | 2001 | NL | This study |
|  | KR478644 | DFO Area 18 (British Columbia, Canada) | 2001 | NL | This study |
|  | KR347078 | DFO Area 18 (British Columbia, Canada) | 2001 | NL | This study |
|  | KR347079 | DFO Area 18 (British Columbia, Canada) | 2001 | NL | This study |
|  | KR347080 | DFO Area 18 (British Columbia, Canada) | 2005 | NL | This study |
|  | KR347084 | DFO area 12 (British Columbia, Canada) | 2013 | N/A | This study |
|  | KR347088 | Hatchery (British Columbia, Canada) | 2013 | N/A | This study |
|  | KR781117 | DFO area 12 (British Columbia, Canada) | 2013 | NL | This study |
|  | KR781118 | DFO area 12 (British Columbia, Canada) | 2013 | NL | This study |
|  | KR558677 | DFO area 12 (British Columbia, Canada) | 2013 | NL | This study |
|  | KR558678 | DFO area 12 (British Columbia, Canada) | 2013 | NL | This study |
|  | KR558679 | DFO area 12 (British Columbia, Canada) | 2013 | NL | This study |
|  | KR558680 | DFO area 12 (British Columbia, Canada) | 2013 | NL | This study |
|  | KR558681 | DFO area 12 (British Columbia, Canada) | 2013 | NL | This study |
|  | KR558682 | DFO area 12 (British Columbia, Canada) | 2013 | NL | This study |
|  | KR558683 | DFO area 12 (British Columbia, Canada) | 2013 | NL | This study |
|  | KR558684 | DFO area 12 (British Columbia, Canada) | 2013 | NL | This study |
|  | KR558685 | DFO area 12 (British Columbia, Canada) | 2013 | NL | This study |
|  | KR347089 | Hatchery (British Columbia, Canada) | 2013 | NL | This study |
|  | KR347090 | Hatchery (British Columbia, Canada) | 2013 | NL | This study |
|  | KR347091 | Hatchery (British Columbia, Canada) | 2013 | NL | This study |
|  | KR347092 | Hatchery (British Columbia, Canada) | 2013 | NL | This study |
|  | KR347100 | Hatchery (British Columbia, Canada) | 2013 | N/A | This study |
|  | KR347101 | Hatchery (British Columbia, Canada) | 2013 | N/A | This study |
|  | KR347094 | Hatchery (British Columbia, Canada) | 2013 | N/A | This study |
|  | KR872637 | Hatchery (British Columbia, Canada) | 2014 | N/A | Garver K. (pers. Comm.) |
|  |  | Hatchery (British Columbia, Canada) | 2014 | N/A | Garver K. (pers. Comm.) |
|  |  | Hatchery (British Columbia, Canada) | 2014 | N/A | Garver K. (pers. Comm.) |
|  |  | Hatchery (British Columbia, Canada) | 2014 | N/A | Garver K. (pers. Comm.) |
|  |  | Hatchery (British Columbia, Canada) | 2014 | N/A | Garver K. (pers. Comm.) |
|  |  | Hatchery (British Columbia, Canada) | 2014 | N/A | Garver K. (pers. Comm.) |
|  |  | Hatchery (British Columbia, Canada) | 2014 | N/A | Garver K. (pers. Comm.) |
|  |  | Hatchery (British Columbia, Canada) | 2014 | N/A | Garver K. (pers. Comm.) |
|  | KR347102 | Hatchery (British Columbia, Canada) | 2013 | N/A | This study |
|  | KR347095 | Hatchery (British Columbia, Canada) | 2013 | N/A | This study |
|  | KR347096 | Hatchery (British Columbia, Canada) | 2013 | N/A | This study |
|  | KR347097 | Hatchery (British Columbia, Canada) | 2013 | N/A | This study |
|  | KR347098 | Hatchery (British Columbia, Canada) | 2013 | N/A | This study |
|  | KR347109 | DFO area 12 (British Columbia, Canada) | 2014 | N/A | This study |
|  | KR347085 | DFO area 7 (British Columbia, Canada) | 2013 | NL | This study |
|  | KR347086 | DFO area 7 (British Columbia, Canada) | 2013 | NL | This study |
|  | KR347087 | DFO area 7 (British Columbia, Canada) | 2013 | NL | This study |
|  | KC473452 | western Canada  Harvest | 2012 | N/A | Kibenge et al. (2013) |
|  | KC473454 | western Canada  Harvest | 2012 | N/A | Kibenge et al. (2013) |
|  | KR347112 | DFO area 27 (British Columbia, Canada) | 2014 | N/A | This study |
|  | KR347113 | DFO area 27 (British Columbia, Canada) | 2014 | N/A | This study |
|  | KR347082 | DFO area 7 (British Columbia, Canada) | 2013 | NL | This study |
|  | KR347115 | Quinsam Hatchery (British Columbia, Canada) | 2014 | N/A | This study |
|  | KR347110 | Quinsam Hatchery (British Columbia, Canada) | 2014 | N/A | This study |
|  | KR347111 | Quinsam Hatchery (British Columbia, Canada) | 2014 | N/A | This study |
|  | KR347093 | Quinsam Hatchery (British Columbia, Canada) | 2013 | N/A | This study |
|  | KR347103 | Quinsam Hatchery (British Columbia, Canada) | 2013 | N/A | This study |
|  | KR347104 | Quinsam Hatchery (British Columbia, Canada) | 2013 | N/A | This study |
|  | KR347099 | Quinsam Hatchery (British Columbia, Canada) | 2013 | N/A | This study |
|  | KR347105 | Quinsam Hatchery (British Columbia, Canada) | 2013 | N/A | This study |
|  | KR347106 | Quinsam Hatchery (British Columbia, Canada) | 2013 | N/A | This study |
|  | KR347107 | Quinsam Hatchery (British Columbia, Canada) | 2013 | N/A | This study |
|  | KR347081 | DFO area 127 (British Columbia, Canada) | 2013 | N/A | This study |
|  | KR347083 | DFO area 127 (British Columbia, Canada) | 2013 | NL | This study |
|  | KR478633 | Columbia River (Washington State, US) | 2012 | N/A | This study |
|  | KR478634 | Columbia River (Washington State, US) | 2012 | N/A | This study |
|  | KR478637 | Columbia River (Washington State, US) | 2014 | N/A | This study |
|  | KR478639 | Columbia River (Washington State, US) | 2014 | N/A | This study |
|  | KR478636 | Columbia River (Washington State, US) | 2014 | N/A | This study |
| P13Type8 | KC473453 | Western Canada  Harvest | 2012 | N/A | Kibenge et al. (2013) |
|  | KC795601 | Western Canada  Harvest | 2012 | N/A | Kibenge et al. (2013) |
|  | KC795600 | Western Canada  Harvest | 2012 | N/A | Kibenge et al. (2013) |
|  | KC795599 | Western Canada  Harvest | 2012 | N/A | Kibenge et al. (2013) |
| P13Type9 | KR872635 | DFO area 124 (British Columbia, Canada) | 2013 | N/A | Garver K. (pers. Comm.) |
|  | KR478638 | Columbia River (Washington State, US) | 2014 | N/A | This study |
|  | KR478635 | Columbia River (Washington State, US) | 2014 | N/A | This study |
|  | KR872636 | DFO area 124 (British Columbia, Canada) | 2013 | N/A | Garver K. (pers. Comm.) |
|  | KR478641 | Copper River (Alaska, US) | 2013 | NL | This study |
|  | KR478640 | Copper River (Alaska, US) | 2013 | NL | This study |
